# Separation of transcriptional repressor and activator functions in *Drosophila* HDAC3

**DOI:** 10.1242/dev.201548

**Published:** 2023-08-02

**Authors:** Min Tang, Isabel Regadas, Sergey Belikov, Olga Shilkova, Lei Xu, Erik Wernersson, Xuewen Liu, Hongmei Wu, Magda Bienko, Mattias Mannervik

**Affiliations:** ^1^ Department of Molecular Biosciences, The Wenner-Gren Institute, Stockholm University, 10691 Stockholm, Sweden; ^2^ Department of Biochemistry and Molecular Biology, University of South China, 421001 Hengyang, China; ^3^ Department of Biosciences and Nutrition, Karolinska Institutet, 14183 Huddinge, Sweden; ^4^ Department of Medical Biochemistry and Biophysics, Karolinska Institutet, 17165 Stockholm, Sweden; ^5^ Science for Life Laboratory, 17165 Stockholm, Sweden

**Keywords:** HDAC3, Histone deacetylase, Chromatin, Transcription, Embryo development, *Drosophila*

## Abstract

The histone deacetylase HDAC3 is associated with the NCoR/SMRT co-repressor complex, and its canonical function is in transcriptional repression, but it can also activate transcription. Here, we show that the repressor and activator functions of HDAC3 can be genetically separated in *Drosophila*. A lysine substitution in the N terminus (K26A) disrupts its catalytic activity and activator function, whereas a combination of substitutions (HEBI) abrogating the interaction with SMRTER enhances repressor activity beyond wild type in the early embryo. We conclude that the crucial functions of HDAC3 in embryo development involve catalytic-dependent gene activation and non-enzymatic repression by several mechanisms, including tethering of loci to the nuclear periphery.

## Teaser

**Highlighted Article:** Amino acid substitutions in a histone deacetylase demonstrate catalytic-dependent gene activation and non-enzymatic gene repression as crucial for *Drosophila* embryo development.

## INTRODUCTION

Precise regulation of gene expression is essential for cell specification and embryo development. Gene regulation largely relies on transcription factors that recruit co-regulators to facilitate association of RNA polymerase II with promoters, in part by modulating the chromatin structure (Cramer, 2019). Many co-regulators are associated with enzymatic activities that can modify histones and non-histone substrates, and protein acetylation and deacetylation in particular are intimately linked to gene regulation (Verdin and Ott, 2015). The histone deacetylase (HDAC) family of enzymes can be divided into four classes based on their catalytic mechanism and sequence homology (Seto and Yoshida, 2014). The class I HDACs include HDAC1, HDAC2, HDAC3 and HDAC8. HDAC3 associates with nuclear receptor co-repressor 1 (NCoR1 or NCoR) and silencing mediator of retinoic acid and thyroid hormone receptor (SMRT; also known as NCoR2), which are both nuclear receptor co-repressors (Lonard and O'Malley, 2007). TBL1X, TBL1XR1 and GPS2 are also subunits of these complexes. In *Drosophila*, the NCoR/SMRT homolog is called SMRTER and TBL1X/TBL1XR1 is known as Ebi. HDAC3 is unique among class I HDACs, as its catalytic function requires physical interaction with a conserved domain in NCoR and SMRT, the deacetylase-activating domain (DAD) (Guenther et al., 2001; Codina et al., 2005). The HDAC3-DAD interaction and HDAC activity is regulated by inositol tetraphosphate (Watson et al., 2012; Millard et al., 2013).

HDAC3 is involved in a diverse set of developmental, physiological and metabolic processes through different molecular mechanism (Emmett and Lazar, 2019). In the mouse liver, HDAC3 represses metabolic and circadian genes through histone deacetylation (Knutson et al., 2008; Kim et al., 2018), whereas in oligodendrocyte precursors the transcription factor STAT3 is inactivated by deacetylation (Zhang et al., 2016). By contrast, in brown adipose tissue HDAC3 activates expression of the uncoupling protein 1 gene by deacetylating the co-activator protein peroxisome proliferator-activated receptor gamma co-activator 1 alpha (PGC1α) (Emmett et al., 2017). It can also activate neural genes in forebrain neurons by deacetylating the transcription factor FOXO3 (Nott et al., 2016).

Although global deletion of HDAC3 in mice is embryonic lethal owing to gastrulation defects (Bhaskara et al., 2008), mice with mutations in the DAD of both NCoR and SMRT are born at the expected Mendelian ratios, despite the lack of detectable HDAC3 enzymatic activity (You et al., 2013). Furthermore, HDAC3 represses cardiomyocyte differentiation by non-enzymatic mechanisms. It silences cardiac linage genes through targeting to the nuclear lamina and the formation of lamina-associated domains associated with heterochromatin and H3K9me2 (Poleshko et al., 2017). In addition, the non-enzymatic activity of HDAC3 represses transforming growth factor-β1 (TGFβ1) signalling by recruiting the H3K27 methyltransferase polycomb repressive complex 2 (PRC2) to prevent the development of cardiac fibrosis (Lewandowski et al., 2015). HDAC3 is also necessary for recruitment of PRC2 and silencing of the X-chromosome for dosage compensation in females (McHugh et al., 2015), and for male fertility and stratification of the embryonic epidermis independent of its enzymatic activity (Szigety et al., 2020; Yin et al., 2021). These observations suggest that HDAC3 also has important non-enzymatic functions.

In *Drosophila*, HDAC3 controls imaginal disc and body size through H4K16 deacetylation (Zhu et al., 2008; Lv et al., 2012), and contributes to repressor activity of the Snail transcription factor in the early embryo (Qi et al., 2008). HDAC3 also cooperates with the SWI/SNF chromatin remodelling Brahma complex to prevent dedifferentiation of larval brain intermediate neural progenitors into neuroblasts (Koe et al., 2014). In larval salivary glands, HDAC3 is a positive regulator of the *Hsp70* gene (Achary et al., 2014). However, the molecular functions of HDAC3 in embryo patterning remain unknown.

In order to dissect these functions, we generated HDAC3 point mutants modelled on mammalian HDAC3. Tyrosine 298 in mammalian HDAC3 is located within the active site and required for catalytic activity, whereas K25 is important for interaction with the DAD (Lombardi et al., 2011; Watson et al., 2012; Sun et al., 2013). A K25A mutation disrupts the DAD interaction and reduces, but does not completely eliminate, binding to full-length NCoR (Sun et al., 2013; You et al., 2013). As other HDACs do not interact with NCoR/SMRT, HDAC3 was mutated in four potential interaction clusters that are divergent between HDAC3 and HDAC1, and named HDAC3 with enzyme and binding activities inactivated (HEBI), because the HEBI mutant abolished the interaction with NCoR/SMRT (Sun et al., 2013). In this work, we made corresponding mutants in endogenous and transgenic *Drosophila* HDAC3 and investigated their effect on survival and embryo patterning. Interestingly, we found that the activator and repressor functions of HDAC3 could be separated by these mutations.

## RESULTS

### HDAC3 catalytic activity is required for *Drosophila* viability

**Fig. 1.**
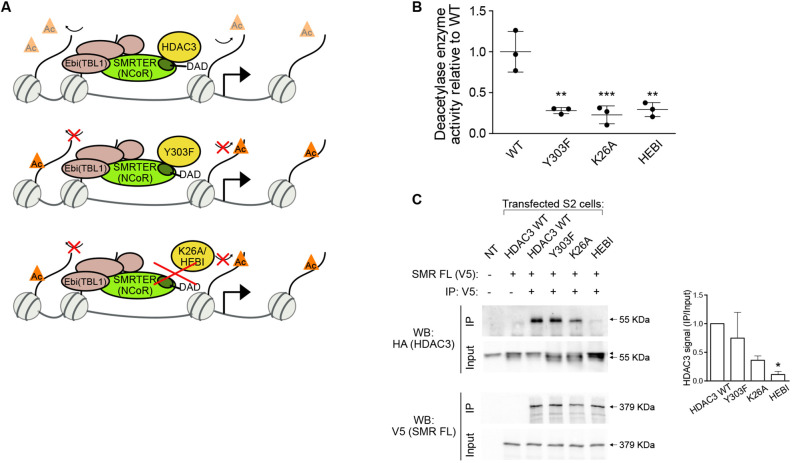
**Impaired catalytic activity of mutant HDAC3 proteins.** (A) Scheme depicting wild-type (WT) and mutant (Y303F, K26A and HEBI) *Drosophila* HDAC3, their deacetylase capacity and interaction with SMRTER (NCoR). (B) Quantification of HDAC3 deacetylase activity after immunoprecipitation from S2 cells and normalization of protein levels by western blot. (C) Western blots following co-immunoprecipitation experiments in S2 cells transfected with V5-tagged SMRTER (SMR) and HA-tagged WT or mutant HDAC3. The graphs show the mean HA-HDAC3 levels in the co-immunoprecipitation over the input. Arrowhead indicates a non-specific band. One-way ANOVA with post-hoc Tukey was applied to compare HDAC3 WT with Y303F, K26A and HEBI. *P<0.05, **P<0.01, ***P<0.001. *n*=2-3, error bars denote s.d.

To investigate the functions of *Drosophila* HDAC3 in development, we generated three mutants that are expected to reduce its catalytic activity (Fig. 1A; Fig. S1A-C). The Y303F, K26A and HEBI mutants are modelled on mammalian HDAC3, where the corresponding Y298F, K25A and HEBI substitutions were shown to interfere with catalysis, reduce the interaction with the SMRT/NCoR DAD and eliminate the interaction with full-length SMRT/NCoR, respectively (Sun et al., 2013). We cloned HA-tagged wild-type (WT) and mutant *Drosophila* HDAC3 genomic regions containing the endogenous promoter and transfected these constructs into S2 cells. After immunoprecipitation and elution, an HDAC assay was performed and deacetylase activity calculated after normaliation of protein levels by western blot (Fig. 1B; Fig. S1D). The Y303F, K26A and HEBI mutants showed a comparable reduction in catalytic activity compared with WT HDAC3. Next, we investigated whether the HDAC3 mutants could interact with SMRTER. We co-transfected HA-tagged HDAC3 and a V5-tagged full-length SMRTER (Fig. 1C). As expected, binding of the Y303F mutant to SMRTER was not greatly affected, K26A weakly reduced binding and the HEBI mutant strongly reduced the interaction with SMRTER. This indicates that reduced catalytic activity of HEBI is likely due to decreased interaction with SMRTER, whereas the K26A mutation may additionally disrupt catalytic activity by some other mechanism.

In order to explore catalytic and other possible functions for HDAC3 *in vivo*, we used homologous recombination to replace the HDAC3 transcription unit with a mini-*white* gene flanked by attP sites (Fig. S2A). The resulting flies, ΔHDAC3, are homozygous lethal and die during the pupal stage. However, 59% survive to the third instar larval stage (Table 1). It appears that the maternal contribution of WT HDAC3 allows for survival of homozygous mutant embryos and larvae to this stage. We then used a recombination-mediated cassette exchange (RMCE)-based approach to re-introduce either WT or the Y303F, K26A and HEBI mutants into the endogenous locus (Fig. S2A-C). Although the WT-replaced flies (HDAC3^WT^) developed into viable adults and 95% made it to third instar larvae, all three catalytically deficient mutants were homozygous lethal. Although many ΔHDAC3 and HEBI mutant animals reached the third instar stage, fewer Y303F or K26A homozygous animals did (Table 1). This indicates that second site mutations on the Y303F and K26A chromosomes were inadvertently introduced during RMCE. Consistent with this notion, a larger fraction of trans-heterozygous combinations of ΔHDAC3 with Y303F or K26A survived to the third instar stage than the homozygous point mutants (Table 1), but they did not survive to adulthood. Western blots from 3-day-old larvae showed a 50% reduction of HDAC3 protein in heterozygous flies (Δ/HDAC3^WT^) compared with a WT (*w^1118^*) strain (Fig. S2D). Similar levels were found in K26A trans-heterozygous mutants, whereas the Y303F and HEBI mutants had reduced HDAC3 protein levels. Some HDAC3 protein, presumably from the maternal load, remained in ΔHDAC3 homozygous larvae (Fig. S2D). This shows that the failure of K26A mutants to survive to adulthood cannot be explained by reduced protein expression, and therefore that the catalytic activity of HDAC3 is essential for viability.

**Table 1.**
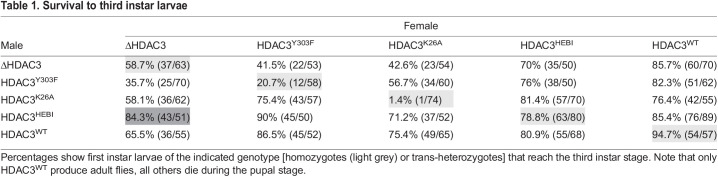
Survival to third instar larvae

Animals from mothers with WT HDAC3 survived better than those from mutant mothers (Table 1, compare column 2 with column 6), showing that the maternal contribution influences their survival. The nature of the zygotic contribution is also important, as animals from ΔHDAC3 heterozygous mothers survived better with a WT-replaced chromosome (65%) than with Y303F (36%) or K26A (58%) (Table 1). Surprisingly, those with a paternal HEBI contribution survived better (84%) than those with a WT chromosome (Table 1, dark gray shading), despite poor expression of the HEBI mutant protein (Fig. S2D). Thus, zygotic HEBI expression rescues viability better than WT HDAC3.

### HDAC3 catalytic function is dispensable for embryonic development

**Fig. 2.**
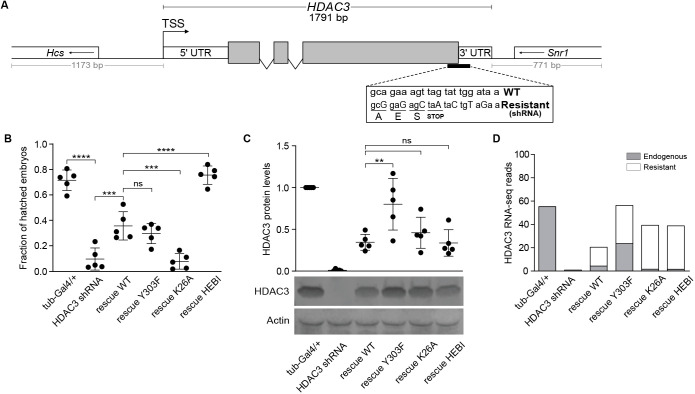
**HDAC3^K26A^ disrupts but HDAC3^HEBI^ supports embryonic development.** (A) Schematic of a HDAC3 shRNA-resistant rescue transgene under the control of its endogenous promoter. Wild-type and shRNA-resistant sequences are shown in the rectangle. (B) Graph showing the fraction of control (tub-Gal4/+), maternal shRNA-mediated HDAC3 knockdown (HDAC3 shRNA) and shRNA-resistant (rescue WT, rescue Y303F, rescue K26A and rescue HEBI) embryos that hatch into first instar larvae. (C) Western blot and quantification showing the levels of HDAC3 protein normalized to Actin in HDAC3 shRNA and shRNA-resistant rescue embryos relative to control (tub-GAL4/+). (D) Endogenous and shRNA-resistant HDAC3 reads from RNA-seq in control (tub-GAL4/+), HDAC3 shRNA and shRNA-resistant rescue embryos. One-way ANOVA with post-hoc Tukey was applied to compare genotypes. **P<0.01, ***P<0.001, ****P<0.0001. ns, non-significant. Error bars indicate s.d.

As the maternal contribution of HDAC3 in ΔHDAC3 homozygous animals supports development to the larval/pupal stage, we needed an alternative method to deplete it in early embryos. We therefore used short hairpin RNA (shRNA)-mediated maternal knockdown of HDAC3 to understand its function in embryo development. We further generated shRNA-resistant rescue transgenes under control of the endogenous promoter and integrated them all at the same genomic landing site (Fig. 2A). As can be seen in Fig. 2B, maternal shRNA-mediated HDAC3 knockdown using the α-tubulin-Gal4VP16 driver results in a failure of most embryos to hatch into larvae. Introduction of one copy of a WT shRNA-resistant transgene partially rescued this phenotype (Fig. 2B). A western blot analysis showed an almost complete absence of HDAC3 protein in shRNA-depleted embryos and incomplete restoration of HDAC3 protein expression in rescued embryos, which may explain the partial rescue (Fig. 2C). We then introduced the point mutations that disrupt HDAC3 catalytic activity in the shRNA-resistant rescue transgenes. The Y303F mutant rescued the hatching phenotype to the same extent as the WT transgene, whereas the K26A mutant failed to rescue (Fig. 2B). Surprisingly, the HEBI mutant rescued even better than the WT transgene (Fig. 2B). The western blot shows that the K26A and HEBI proteins are expressed at levels similar to that of the WT transgene, whereas Y303F is more abundant (Fig. 2C). We performed RNA-seq from these embryos, which allowed us to distinguish between RNA expressed from the endogenous HDAC3 locus and RNA from the shRNA-resistant transgenes. Consistent with the efficient depletion of HDAC3 protein, there is very little HDAC3 RNA in shRNA-treated embryos (Fig. 2D). In embryos from mothers that also have the WT shRNA-resistant transgene, HDAC3 levels are only partially restored and most of this RNA is derived from the transgene. Unexpectedly, in Y303F embryos there is both RNA derived from the shRNA-resistant transgene as well as plenty of RNA derived from the endogenous HDAC3 locus (Fig. 2D). This indicates that the Y303F protein feedbacks on the shRNA-mediated knockdown of HDAC3, and explains why the HDAC3 protein level is higher in these embryos than in WT rescued embryos (Fig. 2C). We speculate that this is due to genetic compensation, which can also be observed in other organisms (El-Brolosy and Stainier, 2017). It also suggests that the reason why Y303F rescues the hatching phenotype to the same extent as the WT transgene is because WT HDAC3 protein is produced in these Y303F-rescued embryos (Fig. 2B). By contrast, in K26A embryos only mutant RNA is produced and the protein levels equal those in WT rescued embryos, but these embryos fail to hatch (Fig. 2B-D). This shows that the K26 residue is essential for embryo development. However, the RNA-seq cannot explain why the HEBI mutant rescues better than WT, as only shRNA-resistant RNA is detected (Fig. 2D). Thus, despite a loss in SMRTER interaction and catalytic activity (Fig. 1), the HEBI mutant supports embryonic development. Taken together, these results show that HDAC3 is required for embryo development, that catalytic activity is dispensable (as the HEBI mutant rescues the hatching phenotype), but that the K26A mutation disrupts the essential HDAC3 embryonic function.

### Unique functions for HDAC3 amino acids in embryonic patterning

**Fig. 3.**
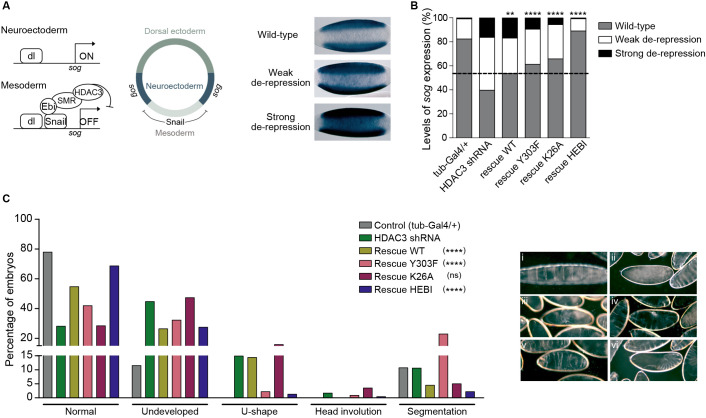
**Unique functions of HDAC3 mutants in embryonic patterning.** (A) The *sog* gene is activated by Dorsal protein (dl) in the neuroectoderm of early embryos and repressed by Snail with the help of Ebi/SMR/HDAC3 in the mesoderm. *In situ* hybridization shows the wild-type (WT) expression pattern of *sog* restricted to the neuroectoderm, as well as weak and strong de-repression in the mesoderm. Ventral views of 2- to 4-h-old embryos with anterior to the left. (B) Graph shows the percentage of embryos with WT, weak and strong de-repression of *sog* expression in control (tub-GAL4/+, *n*=210), HDAC3 shRNA (*n*=224) and shRNA-resistant rescue genotypes (rescue WT, *n*=261; rescue Y303F, *n*=253; rescue K26A, *n*=199; rescue HEBI, *n*=254). Phenotypic ratios were tested to see whether they differed significantly between HDAC3 shRNA knockdown embryos and embryos containing rescue transgenes using pairwise χ2 tests. **P<0.01, ****P<0.0001. Note that Y303F also expresses WT endogenous HDAC3. (C) Left: cuticle phenotypes in the depicted genotypes (tub-GAL4/+, *n*=122; HDAC3 shRNA, *n*=235; rescue WT, *n*=201; rescue Y303F, *n*=227; rescue K26A, *n*=317; rescue HEBI, *n*=226). Pairwise χ2 tests were applied to compare the cuticle phenotypes of HDAC3 shRNA knockdown animals with the phenotypes in animals containing rescue transgenes. ****P<0.0001. ns, non-significant. Right: Pictures with representative examples of embryos showing a normal cuticle phenotype (i), undeveloped (ii, iii) and U-shaped (iv), segmentation (v) and head involution (vi) defects.

We have previously demonstrated that specification of mesoderm in the early embryo by the Snail repressor requires the Ebi protein (Qi et al., 2008), a homolog of mammalian TBL1X/TBL1XR1. Although Ebi is part of the SMRTER complex and binds to HDAC3, the catalytic function of HDAC3 in Snail-mediated repression has not been investigated. We therefore collected 2- to 4-h-old embryos and performed whole-mount *in situ* hybridization with a *short-gastrulation* (*sog*) probe (Fig. 3A). Snail protein is present in the mesoderm where it represses *sog* and restricts its expression to the presumptive neuroectoderm (Qi et al., 2008). In HDAC3 shRNA knockdown embryos, *sog* expression was partially de-repressed in the mesoderm, demonstrating that HDAC3 contributes to Snail-mediated repression of *sog* (Fig. 3A,B). The *sog* expression pattern was classified as normal, weak or strong de-repression in the mesoderm (Fig. 3B). In WT rescued embryos, the number of embryos with a normal *sog* pattern increased from 40% to 54%. Remarkably, all three HDAC3 mutant constructs also rescued *sog* expression in the mesoderm (Fig. 3B). This shows that HDAC3 catalytic activity is dispensable for Snail-mediated repression of *sog* transcription.

To further investigate the embryo phenotype we performed cuticle preparations on embryos from the different genotypes. This showed that 45% of HDAC3 shRNA-depleted embryos did not develop cuticle, 28% showed a normal cuticle phenotype, 15% were U-shaped, 10% had segmentation defects and head involution was defective in less than 2% (Fig. 3C). Consistent with the hatching rate phenotype, WT rescued embryos showed an increase in those with a normal cuticle from 28% to 55%. The K26A rescued embryos had very similar phenotypes to the HDAC3 shRNA knockdown embryos, whereas 42% normal cuticle in Y303F rescued embryos was intermediate to knockdown and WT rescue (Fig. 3C). Interestingly, the frequency of segmentation defects increased to 23% in Y303F embryos, despite the presence of some WT HDAC3 in these embryos. This indicates that the Y303F mutation in addition to disrupting catalytic function also interferes with an additional HDAC3 activity that is needed for segmentation, as no other HDAC3 mutant showed this strong segmentation phenotype. In HEBI rescued embryos, 69% had a normal cuticle, more than in WT rescued embryos (Fig. 3C). Although embryos that did not develop cuticle were similar between HEBI and WT rescued, the number of embryos with a U-shaped phenotype was reduced from 14% in WT to 1% in HEBI-rescued embryos (Fig. 3C). The cuticle phenotype analysis shows that the different HDAC3 substitutions generate different embryonic phenotypes, indicating that there are unique functions associated with these amino acids in HDAC3.

### Separation of repressor and activator functions in HDAC3

**Fig. 4.**
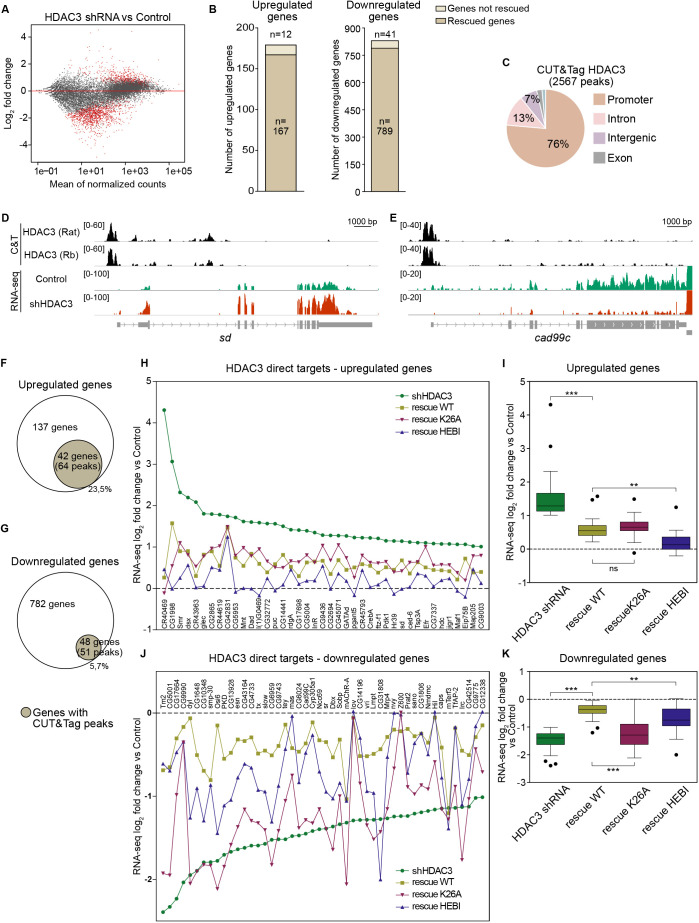
**HDAC3^HEBI^ improves repressor activity and HDAC3^K26A^ impairs HDAC3 activator function in the embryo.** (A) MA plot of differentially expressed genes between HDAC3 shRNA and control (tub-GAL4/+) identified by RNA-seq. (B) Total number of differentially expressed genes between HDAC3 shRNA and control (upregulated genes, 179; downregulated genes, 830; log_2_ fold change ≥1, FDR ≤0.05) and the number of genes rescued by the wild-type (WT) HDAC3 shRNA-resistant transgene (log_2_ FC <1 versus control). (C) Genomic distribution of 2567 CUT&Tag peaks in common between two HDAC3 antibodies in 2- to 4-h-old *w^1118^* WT embryos. (D,E) Genome browser screenshots of one upregulated (*sd*; D) and one downregulated (*cad99c*; E) gene with tracks for HDAC3 CUT&Tag with two different antibodies and for RNA-seq signal (HDAC3 shRNA and control). (F,G) Venn diagrams depicting the number of differentially expressed genes in HDAC3 shRNA embryos with HDAC3 CUT&Tag peaks. (H,J) Gene expression log_2_ fold change of individual HDAC3 direct targets relative to control in HDAC3 shRNA embryos and in embryos rescued by WT or mutant HDAC3. Rescue by Y303F and an outlier in K26A is shown in Fig. S3. The dashed line intersecting 0 on the *y*-axis represents control embryos (Tub-Gal4/+). (I,K) Box-plots showing the average log_2_ fold expression change of HDAC3 direct target genes in HDAC3 shRNA embryos and in embryos rescued by WT or mutant HDAC3 relative to control. The dashed line intersecting 0 on the *y*-axis represents control embryos (Tub-Gal4/+). One-way ANOVA with post-hoc Tukey was applied to compare different genotypes. The middle line in the box plot shows the median and whiskers indicate lower and upper quartiles. **P<0.01, ***P<0.001. ns, non-significant. Error bars show standard deviation.

To gain further insight into the embryonic functions of HDAC3, we generated RNA-seq data from control and HDAC3 shRNA knockdown embryos, and from knockdown embryos with shRNA-resistant WT, Y303F, K26A or HEBI rescue transgenes in four biological replicates (Fig. 4; Fig. S3). Although HDAC3 is believed to function mainly as a repressor, we found that more genes were downregulated (*n*=830, fold change ≥2, FDR 0.05) than upregulated (*n*=179) in knockdown embryos (Fig. 4A,B; Fig. S3A; Table S1). Interestingly, downregulated genes were less strongly expressed in control embryos than most upregulated genes, which were already highly expressed in control embryos and further increased in HDAC3 knockdown embryos (Fig. 4A). The majority of these were rescued to less than a 2-fold change by the WT HDAC3 shRNA-resistant transgene (95% of downregulated and 93% of upregulated genes), demonstrating few off-target effects in shRNA knockdown embryos (Fig. 4B).

As the gene expression changes can be direct or indirect, and as maternal HDAC3 shRNA knockdown also affected maternally contributed embryonic transcripts from the egg (see Table S1), we generated CUT&Tag data with two different HDAC3 antibodies to identify direct embryonic targets. We identified 2567 high confidence HDAC3-specific peaks in common between the two antibodies in 2- to 4-h-old embryos (Fig. S3C), and most of these are located in promoters (76%) and in introns (13%) (Fig. 4C). Genome browser screenshots show examples of upregulated and downregulated genes (Fig. 4D,E). Only a minority of mis-expressed genes in HDAC3 knockdown embryos were bound by HDAC3 at this stage, 42 upregulated (23%) and 48 downregulated (6%) genes (Fig. 4F,G), suggesting that many genes are affected by maternal HDAC3 during oogenesis or are indirect targets. As a control, we performed HDAC3 CUT&Tag in WT and HDAC3 knockdown embryos. In this experiment, fewer WT peaks were detected, but they were largely depleted from knockdown embryos as only 65 out of 1142 peaks remained (Fig. S3D-F). A gene ontology (GO) analysis showed that upregulated genes are involved in reproduction and that many direct targets are transcription factors, whereas downregulated genes are involved in cuticle and muscle development (Table S2). Thus, HDAC3 directly regulates a few genes that are crucial for embryonic development.

We then plotted the log_2_ fold change in expression for each direct target in HDAC3 shRNA embryos and in embryos rescued by WT or mutant HDAC3 (Fig. 4H-K; Fig. S3G-J). Although WT HDAC3 rescued all upregulated genes relative to their expression in HDAC3 shRNA embryos, they did not reach the same level of expression as in control embryos (Fig. 4H). However, consistent with the hatching and cuticle results (Figs 2 and 3), the HEBI mutant rescued most upregulated genes to a larger extent than WT HDAC3 and to levels very close to those in control embryos (Fig. 4H,I). This indicates that HDAC3 represses the majority of genes in the early embryo independently of its catalytic activity and independently of the interaction with SMRTER. By contrast, expression of most downregulated genes is more efficiently rescued by WT than any of the mutant HDAC3s (Fig. 4J,K; Fig. S3H,J). Therefore, catalytic activity is required for activation but not for repression.

Interestingly, although K26A failed to rescue embryonic lethality and the cuticle phenotype, it behaved very similar to WT HDAC3 in rescuing the upregulated genes (Fig. 4H,I). However, it was much poorer than WT at rescuing downregulated target genes (Fig. 4J,K). Together, these results show that the HEBI mutations improve the repressor function of HDAC3, whereas K26A disrupts HDAC3 activator function.

### Targeting to the nuclear lamina or to Polycomb-repressed domains constitutes a minor form of HDAC3-dependent repression in the embryo

**Fig. 5.**
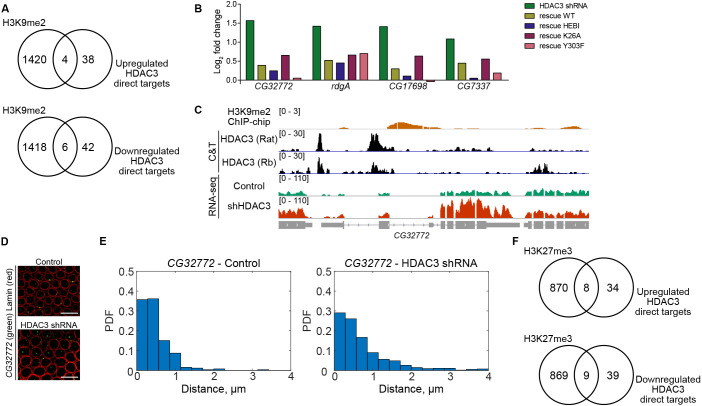
**Catalytic-independent HDAC3 repression in early embryo development.** (A) Number of overlapped peaks between modENCODE embryonic H3K9me2 ChIP-chip data (Roy et al., 2010) and HDAC3 direct target genes. (B) Expression fold change versus control (tub-GAL4/+) in HDAC3 shRNA and in embryos rescued by wild-type (WT) or mutant HDAC3 for upregulated HDAC3 targets that contain H3K9me2 peaks. (C) Genome browser screenshots of the *CG32772* locus with tracks for H3K9me2 ChIP-chip peaks, HDAC3 CUT&Tag with the rat and rabbit (Rb) antibodies and for RNA-seq signal (HDAC3 shRNA and control). (D) Image of DNA FISH in 2- to 4-h-old WT (control) and HDAC3 shRNA embryos using a probe for *CG32772* (green) followed by an immunostaining for Lamin (red). Scale bar: 10 μm. (E) Measurement of the distance of the *CG32772* locus to the nuclear periphery in control and in HDAC3 shRNA embryos, plotted as probability density functions (PDFs) where the bars in each plot sum up to one. (F) Number of overlapped peaks between embryonic H3K27me3 ChIP-seq data (Li et al., 2014) and HDAC3 direct target genes.

To investigate what mechanisms could be involved in the non-catalytic functions of HDAC3, we compared modENCODE embryonic H3K9me2 data with the gene expression changes in HDAC3-depleted embryos (Roy et al., 2010). In mammalian cells, HDAC3 has been shown to maintain some loci located within lamina-associated domains (LADs) close to the nuclear periphery (Poleshko et al., 2017). LADs are decorated with H3K9me2 (van Steensel and Belmont, 2017) and we therefore expected some of the HDAC3 targets that are enriched for this modification to be repressed by this mechanism. We found that four of the upregulated genes and six of the downregulated genes contain H3K9me2 in WT embryos (Fig. 5A). All of these upregulated genes were rescued by both WT and mutant transgenes (Fig. 5B), confirming that they require a non-enzymatic HDAC3 function for their expression. A genome browser screenshot for one of them, *CG32772*, is shown in Fig. 5C. We used DNA fluorescent *in situ* hybridization (FISH) in 2- to 4-h-old embryos for this locus and for *CG17698*, and they were observed to localize to the nuclear periphery in WT embryos (Fig. 5D; Fig. S4A). Interestingly, the loci were found at a larger distance from the periphery in HDAC3 shRNA knockdown embryos (Fig. 5D,E; Fig. S4A,B). By contrast, the *sog* gene locus that lacks H3K9me2 was located further from the periphery in WT embryos and did not shift its location towards the nuclear interior in HDAC3 knockdown embryos (Fig. S4C,D), and was therefore repressed in an alternative catalytic-independent way. From this, we conclude that nuclear lamina targeting with the help of HDAC3 is a mechanism by which ∼10% of HDAC3-repressed genes could be regulated in the embryo.

Another mechanism that has been implicated in HDAC3-mediated repression is recruitment of Polycomb Group (PcG) proteins (Lewandowski et al., 2015). We therefore examined whether HDAC3-regulated genes are associated with H3K27me3 in embryos. This showed that eight of the HDAC3-repressed genes overlapped with H3K27me3 (Fig. 5F). Taken together, our data suggest that most of the HDAC3-target genes in the early embryo are repressed through an uncharacterized mode that does not require catalytic activity, targeting of loci to the nuclear lamina or recruitment of PcG proteins.

## DISCUSSION

The canonical function of HDAC3 is to repress target genes as part of the NCoR/SMRT complex by histone deacetylation. We found that the essential function of HDAC3 in *Drosophila* embryo development involves catalytic-dependent gene activation. A single lysine substitution, K26A, impairs catalytic activity, disrupts embryo hatching and fails to activate target genes in the early embryo. However, it is able to repress most embryonic target genes to a similar extent as WT HDAC3. Although the mechanism by which HDAC3 activates transcription in the embryo remains unclear, studies in mammalian cells have shown that HDAC3 can activate genes in multiple contexts. In brown adipose tissue, HDAC3 associates with estrogen-related receptor alpha (ERRα) and activates the coactivator PGC1α by deacetylation (Emmett et al., 2017). In the forebrain, HDAC3 activates some neuronal genes by its recruitment by MECP2 and deacetylation of the transcription factor FOXO3 (Nott et al., 2016). During activation of macrophages by lipopolysaccharides, HDAC3 is recruited by ATF2 and activates inflammatory genes (Chen et al., 2012; Nguyen et al., 2020). We speculate that also in the *Drosophila* embryo HDAC3 influences the activity of a transcription factor to execute gene activation.

HDAC3 has multiple deacetylase-independent functions in development and physiology. Global deletion of HDAC3 is embryonic lethal, whereas mice with mutations in both the NCoR and SMRT DADs that abolish HDAC3 enzymatic activity are born in expected Mendelian ratios (You et al., 2013). Furthermore, point mutants that abolish catalytic activity can partially rescue HDAC3-dependent phenotypes in the mouse liver (Sun et al., 2013). One mechanism by which HDAC3 represses transcription non-enzymatically is by recruitment of the PRC2 complex and induction of H3K27me3. In the mouse second heart field, HDAC3 silences TGFβ1 expression through this mechanism (Lewandowski et al., 2015). However, we found that only ∼20% of the HDAC3-repressed genes in the *Drosophila* embryo are decorated with H3K27me3.

Tethering genes to the nuclear periphery in LADs is another non-catalytic repression mechanism. In mouse embryonic stem cells, HDAC3 tethers cardiac lineage genes to the nuclear lamina through an interaction with LAD-associated proteins such as emerin (Somech et al., 2005; Demmerle et al., 2012; Zullo et al., 2012; Poleshko et al., 2017). We found that this is one of the mechanisms by which HDAC3 represses a subset of genes in the *Drosophila* embryo as well. However, a majority of repressed genes lack the H3K9me2 mark associated with LADs, and are therefore most likely repressed by an unknown non-enzymatic mechanism.

Interestingly, the HEBI combination of mutations improve the repressor function of HDAC3. These mutations are modelled on mammalian HDAC3 where they were shown to disrupt the interaction with NCoR/SMRT and thereby abolish catalytic activity (Sun et al., 2013). We found that corresponding mutations in *Drosophila* HDAC3 also interfere with the SMRTER interaction and impair catalytic activity. As the HEBI mutant protein is better than WT at rescuing HDAC3 phenotypes, it suggests that a fraction of HDAC3 normally exists outside the SMRTER complex, is enzymatically inactive, but acts as a transcriptional repressor. Recent results in mouse macrophages found that HDAC3 associates with a subset of genes independently of NCoR/SMRT, consistent with a model of HDAC3 function outside this co-repressor complex (Nguyen et al., 2020).

Intriguingly, the enhanced repressor function of HEBI mutant HDAC3 improves survival, both when expressed from an shRNA-resistant transgene and when knocked-in at the endogenous locus. This indicates that HDAC3 has a key developmental role outside of the SMRTER complex. Interestingly, association with the NCoR/SMRT DAD is allosterically regulated by the HDAC3 C terminus, which induces a conformational change that is necessary for the interaction with the DAD (Li et al., 2021a). This explains older results showing that the C-terminus is necessary for catalytic activity (Yang et al., 2002). As the C-terminus can be phosphorylated by several kinases (Tsai and Seto, 2002; Sengupta and Seto, 2004; Zhang et al., 2005, 2021; Seo et al., 2019), it suggests that the HDAC3-DAD interaction is regulated by signalling. Furthermore, a recent study showed that NADPH inhibits HDAC3-DAD complex formation by competing with IP4 for binding to HDAC3 (Li et al., 2021b). Thus, metabolic state and cell signalling regulate the balance between DAD-bound and -unbound HDAC3, which may be a mechanism to control its transcriptional repressor activity.

## MATERIALS AND METHODS

### Molecular cloning

#### HDAC3 knock-in constructs

The donor construct for homologous recombination is based on the vector pP(whiteSTAR) from Choi et al. (2009), in which the multiple cloning sites (MCS) were modified to insert AscI and AgeI sites at the SpeI site, and by insertion of a UAS-Rpr element for negative selection (Huang et al., 2008). The vector was named pP(whiteSTAR)Rpr13. Homologous arms of 4.4 and 3.2 kb in size were PCR amplified from genomic DNA and inserted upstream and downstream of the *white+* marker gene used for positive selection. The left arm amplicon used primers Hdac3LAfor and Hdac3LAAcc65Irev and was cloned into pGEM-T Easy, from which it was released by Acc65I digestion and cloned into the left polylinker Acc65I site in pP(whiteSTAR)Rpr13. Hdac3RAAscIfor and Hdac3RAAgeIrev primers were used to amplify the right arm, cloned into pGEM-T Easy, released with AscI and AgeI and cloned in the right polylinker of pP(whiteSTAR)Rpr13.

A 1.8 kb *HDAC3* genomic region from 263 bp upstream of the HDAC3 translational start site to 66 bp downstream of the HDAC3 open reading frame was PCR amplified with primers pABCHDAC3frw and pABCHDAC3rev and cloned into the Hind III restriction site of pABC (Choi et al., 2009), which flanks the insert with attB-sites. The amino acid substitutions K26A, Y303F or HEBI (K47E, LR160-161TK, EGAQ118-121AAAV, HNH125-127KQA) were introduced into the knock-in construct by PCR amplification of the entire vector using two phosphorylated primers, which have overhangs containing the mutations. The PCR reaction was DpnI-treated and blunt-end ligated to circularize the PCR product into a plasmid. Correct clones were identified by restriction digestion and DNA sequencing.

#### HDAC3 knockdown resistant transgenes

The *HDAC3* genomic region from 1173 bp upstream of the HDAC3 transcription start site (TSS) to 771 bp downstream of the 3′ untranslated region was PCR amplified using primers HDAC3GR_f and HDAC3GR_r. The PCR product was cloned into the XbaI restriction site of the pattB vector (Bischof et al., 2007), resulting in the plasmid pAttB-HDAC3. An miRNA-resistant sequence was introduced into pattB-HDAC3 by PCR amplification of the entire vector using two phosphorylated primers HDAC3 res_f and HDAC3 res_r, which have overhangs containing one half of the miRNA-resistant sequence each. The PCR reaction was DpnI-treated and blunt-ligated to circularize the PCR product into a plasmid. The amino acid substitutions K26A, Y303F or HEBI (K47E, LR160-161TK, EGAQ118-121AAAV, HNH125-127KQA) were introduced into pattB-HDAC3-resistant construct by PCR amplification of the entire vector using two phosphorylated primers, which have overhangs containing the mutations. After DpnI-treatment, clones were identified by restriction digestion and DNA sequencing.

#### HA-tagged HDAC3

A HindIII restriction enzyme site was introduced into the translation termination site of the plasmid pAttB-HDAC3 by PCR amplification of the entire vector using two phosphorylated primers, HDAC3 C Hind III F and HDAC3 C Hind III R, that have overhangs containing the HindIII restriction site. Then two HA and two FLAG-tag sequences were amplified from the pRmHa-3 vector using the primers HDAC3 C tag F and HDAC3 C tag R, and cloned into the HindIII restriction site by Gibson assembly (New England Biolabs, E2611).

#### V5-tagged Smr

The SMRTER (*Smr*) gene contains nine exons and multiple introns, of which three are long. Five pairs of primers were designed to avoid the large introns. The *Smr* genomic region was separated into five fragments and PCR amplified using these primers. Each fragment was cloned one by one into the XbaI restriction site of the pAC 5.1 vector that has a V5 tag, using a Gibson assembly kit (New England Biolabs, E2611). The base A was introduced into each reverse primer to form an XbaI restriction site. The final Smr full-length construct deleted the large introns but contained introns between exons 3-5 and 7-9. Primers are listed in Table S3.

### Fly stocks and generation of knockout, knock-in and miRNA-resistant flies

We used an ‘ends-out’ gene targeting approach modified from Rong and Golic (2000) to delete the *HDAC3* gene, in which the positive selection marker mini-*white*+ is flanked by attP sites and combined with negative selection using the cell death gene *reaper* (*Rpr*). The donor construct pP(whiteSTAR) Hdac3 was injected into the *w^1118^* strain. Established donor lines were mapped and donor construct integrity validated as described in the supplementary materials of Huang et al. (2008). A donor line integrated on the second chromosome was used for homologous recombination. To validate donor construct integrity the donor line was crossed to the *w-* Gal4-trap lines Gal4^477^ or Gal4^221^ that activate Rpr expression in the nervous system that leads to lethality, and to P{70FLP}10 causing mosaic eyes.

Targeting crosses were set exactly as described in Huang et al. (2008). The 6934-hid strain contains hs-hid transgenes on the Y and balancer chromosomes, eliminating all male progeny and those female progeny carrying a hs-hid balancer chromosome after heat shock. Therefore, P{donor}/hs-FLP, hs-I-SceI females are the only genotype that survives. Fifty vials, each containing 30 virgin females of transgenic donor flies were mated with 30-40 males of the 6934-hid strain. The crosses were maintained at room temperature and the flies flipped to fresh vials every 24 h. Eggs in transferred vials were aged at 25°C and were heat shocked at day 2 (24-48 h after egg-laying) and day 3 (48-72 h after egg-laying). Heat shock was carried out at 38°C for 60-90 min in a circulating water bath.

For screening crosses, ten virgin females from the targeting cross were mated with *w; Gal4^477^ w-; TM2/TM6B, Tb* males to eliminate non-targeted events. Flies were flipped every couple of days five times and kept at 25°C. Preliminary candidates were screened by eye colour based on the *white+* marker. For the mapping cross, candidates were crossed with balancer flies individually. The targeting event was confirmed by PCR with primers annealing in the *white+* gene marker and outside of the donor construct.

These knockout flies, ΔHDAC3, where an attP-flanked mini-*white+* gene replaced HDAC3, were used for knock-in of WT or mutant HDAC3 sequences. Plasmid pABC-HDAC3 containing the HDAC3 gene flanked with attB-sites was used for phiC31-mediated site-specific integration to substitute mini-*white*. Females expressing phiC31 in the germline, *y^1^ M{vas-int.Dm}ZH-2A w*, were crossed with ΔHDAC3 flies and the embryos injected with pABC-HDAC3. Progeny were screened for loss of mini-*white* and PCR was used to validate the integration using the primers HDAC3m knockin test F and HDAC3m knockin test R.

Bloomington Drosophila Stock Center stock #34778 (*y^1^ sc v^1^ sev^21^; P{TRiP.HMS00087}attP2*) containing an shRNA encoding an HDAC3 miRNA was used to knockdown HDAC3 in the early embryo. The *w; P{w[+mC]=matalpha4-GAL-VP16}V2H* strain (referred to as tub-Gal4) was used as maternal Gal4 driver. The pattB-HDAC3 WT and resistant transgenic constructs were all inserted at the attP40 landing site on the second chromosome and combined with the shRNA shmiRNA line (#34778) on chromosome 3 and made homozygous using double balancer flies. These flies were then crossed with the tub-Gal4 driver to generate females where knockdown occurs in the female germline.

### Animal survival

The HDAC3 knockout strain and all knock-in mutants were balanced with *TM6 Tb, Dfd-YFP*. The different mutants were selected and crossed to each other in food bottles for 2-4 days before being transferred to embryo collection cages. Embryos were collected from the cages on fruit juice agar plates supplemented with yeast and raised until first instar larva. The number of YFP-negative first instar larvae that failed to develop to third instar larvae were counted.

### Embryo survival

At least 50 virgin homozygous tub-Gal4 females were placed in food bottles together with homozygous males carrying the shRNA and shRNA-resistant transgene of interest. The progeny, F1 females and males, were allowed to mate with each other in the food bottles for 2-4 days before being transferred to embryo collection cages. Embryos were collected from the cages on fruit juice agar plates supplemented with yeast. The number of embryos that could hatch was counted.

### Cuticle preparation and RNA *in situ* hybridization

For cuticle preparations, the embryos were aged, dechorionated in bleach, transferred to microscopic slides, cleared in lactic acid at 65°C and cuticles examined using dark-field microscopy (Wieschaus and Nüsslein-Volhard, 1988). The whole-mount embryo RNA *in situ* hybridization protocol was modified from Qi et al. (2008). Probes against *sog* were generated by PCR amplification with primers where Sp6 overhangs were added, and used for *in vitro* transcription. The *sog* derepression and cuticle phenotypes were counted unaware of the genotype.

### DNA fluorescent *in situ* hybridization

We selected 12 kb gene regions centred at the TSS and subdivided them into six different fragments. DNA fragments of 1.2-1.7 kb covering 12 kb were PCR amplified from genomic DNA and labelled with Alexa Fluor 488 dye FISH Tag DNA kit (F32947, Invitrogen). DNA FISH was performed in 2- to 4-h-old embryos based on the protocol in Bantignies and Cavalli (2014), and the nuclear membrane marked with anti-lamin Dm0 antibody (Developmental Studies Hybridoma Bank, ADL84.12, 1:1000) followed by a Cy5-labelled secondary antibody (Jackson ImmunoResearch, 715-175-151, 1:800). For the *sog* probe, a Snail antibody (1-244; a kind gift from Yutaka Nibu, Nagoya University, Japan) was used to label the mesoderm in order to distinguish it from the neuroectoderm region.

Samples were imaged on a Nikon Ti-E microscope equipped with a sCMOS camera (Andor Technology) using a CFI Plan Apochromat Lambda 100× Oil objective (Nikon). Nuclei were segmented by first applying a median filter of size 13×13×5 pixels to suppress noise and small local variations. Then the images were segmented using thresholds calculated by Otsu's method. Finally a morphological dilation was applied using a 5×5×3 structuring element to grow the segmentation masks slightly in order to fully cover the lamin staining. Dots were detected in AF488 and Cy5 channels using the Difference of Gaussians method. Locations were further refined by replacing each pixel coordinate by the local 3D centre of mass. For Cy5 this gave us a quite dense sampling of the lamin staining. For each point in a nucleus we reported the shortest distance to lamin (represented by any dot in Cy5) after rescaling the pixel coordinates by the pixel size. Alternatively, nuclei were segmented by manually placing five or more control points of a circular cubic spline for each nucleus. This was performed on top of a maximum projection over *z*, showing a composite colour image of the three channels.

For each nucleus the segmentation mask was rasterized to a 2D image. The distance transform was used to define a distance map with respect to the segmentation contours (lamin) for each nucleus. The distances were normalized from 0 to 1 (centre of the nucleus) by dividing by the maximum value per nucleus. For the plots in Fig. 5 and Fig. S4 we presented the data as probability density functions (PDFs) where the bars in each plot sum up to one.

### HDAC assay

*Drosophila* S2 cells (Drosophila Genomics Resource Center, stock #6) were transfected with HA-tagged HDAC3, then lysed in lysis buffer (20 mM Tris pH 7.5, 150 mM NaCl, 1% NP40) containing protease inhibitors. Lysate was added to pre-washed HA-conjugated beads (Pierce anti-HA magnetic beads, #88837, Thermo Fisher Scientific) and incubated at 4°C overnight. Immunoprecipitates were washed three times with TBST buffer (20 mM Tris pH 7.5, 150 mM NaCl, 0.1% Tween 20) and eluted with 90 μl HA peptide solution (final concentration 2 mg/ml). We used 60 μl elution buffer in a HDAC assay kit (Active Motif, #56200) following the manufacturer's instructions. We separated 12 μl elution buffer on an 10% SDS-PAGE gel and transferred to a PVDF membrane, and then probed with a rat anti-HDAC3 antibody diluted 1:1000 (Qi et al., 2008), which was detected using IRDye^®^ 680RD secondary antibodies (LI-COR, 926-68076, 1:20,000) using a LI-COR machine to quantify HA-tagged HDAC3. The enzyme activity was normalized to HA-tagged HDAC3 protein concentration.

### Co-immunoprecipitation

Co-immunoprecipitation was performed by preparing protein extracts from S2 cells transfected with HA-tagged HDAC3 and V5-tagged Smr. V5 antibody (Invitrogen, R96025) was added to samples for immunoprecipitation with protein A/G magnetic beads. Protein samples were separated on an 10% SDS-PAGE gel, transferred to a PVDF membrane and probed with an HA antibody (3F10, Roche, 1:500) followed by an HRP-coupled secondary antibody (Dako, P0450, 1:2000) and enhanced chemiluminescence (ECL; GE Healthcare).

### Western blot

HDAC3 mutant larvae, obtained from 0- to 4-h-old embryos and raised at 25°C for 3 days, and 2- to 4-h-old knockdown embryos were harvested and directly lysed in RIPA buffer. Protein was separated by 10% SDS-PAGE gel and transferred to a PVDF membrane. The membrane was then probed with rat anti-HDAC3 (Qi et al., 2008; 1:1000) and anti-actin (Invitrogen, MA5-11869, 1:400) antibodies, followed by horseradish peroxidase conjugated secondary antibodies (Dako, P0450 and P0447, 1:2000). Protein bands were visualized using ECL reagents (GE Healthcare). The protein band intensities were quantified using ImageJ Software using actin as a loading control.

### RNA-seq

HDAC3 knockdown and rescued 2- to 4-h-old embryos were collected in biological quadruplicates and total RNA was extracted using Trizol (Invitrogen). For each biological replicate, 20 μl of embryos were collected. Libraries were prepared using an Illumina TruSeq kit at the Bioinformatics and Expression Analysis (BEA) facility at the Karolinska Institutet, Sweden, and sequenced on a NextSeq 500.

### CUT&Tag

Wild-type (*w^1118^*) and HDAC3 shRNA embryos were collected on apple juice agar plates supplemented with yeast for 2 h and aged an additional 2 h. Following dechorionation, crude nuclei were isolated from ∼200 embryos by homogenization using a glass douncer and a pestle in nuclear extraction buffer (20 mM HEPES pH 7.9, 10 mM KCl, 0.5 mM spermidine, 0.1% Triton X-100, 20% glycerol) (Hainer and Fazzio, 2019) and centrifugation at 700 ***g*** for 10 min. The obtained nuclear pellet was resuspended in 200 µl of nuclear extraction buffer and incubated with 20 µl of BioMag Plus Concanavalin A beads (Polysciences) for 10 min at 4°C. The resulting nuclei/beads complex was incubated with 2 µl of primary antibody (rat or rabbit anti-HDAC3) in 200 µl of antibody buffer (Kaya-Okur et al., 2019) overnight at 4°C. Afterwards, the experimental procedure was followed as previously described (Kaya-Okur et al., 2019), using pA-Tn5 produced at the Protein Science Facility, Karolinska Institutet, and assembled with Mosaic End double-stranded (MEDS) oligonucleotide adaptors in the lab, according to Kaya-Okur et al. (2019). The loaded pA-Tn5 was used at a 1:500 dilution. Tagmented DNA was purified with the DNA Clean & Concentrator-5 kit (D4014, Zymo Research) and amplified using PhusionHigh-Fidelity PCR Master Mix with GC Buffer (New England Biolabs), with 1.25 mM of Universal i5 primer and 1.25 mM of uniquely barcoded i7 primers for each sample (Buenrostro et al., 2015). PCR conditions were: 72°C for 5 min; 98°C for 30 s; thermocycling for 13 cycles at 98°C for 10 s and 63°C for 10 s; and one final step at 72°C for 1 min. Following amplification, libraries were purified with Agencourt AMPure XP beads (Beckman Coulter) using a 1.1:1 volume of beads to sample. Libraries were sequenced (paired-end, 2×75 bp) in the NextSeq 550 Sequencing platform (Illumina) at BEA core facility.

### Bioinformatics

#### Differential expression analysis

RNA-seq reads were mapped to the *Drosophila melanogaster* (dm6) genome assembly using STAR and default parameters (Dobin et al., 2013). RNA-seq differential expression analysis of HDAC3 shRNA or shRNA-resistant rescue genotypes (rescue WT, rescue Y303F, rescue K26A, rescue HEBI) versus control (tub-GAL4/+) was performed using DEseq2 (Love et al., 2014) with count tables generated from featureCounts (Liao et al., 2014). From the differentially expressed genes that were identified, we selected the ones with FDR<5% and fold change ≥2 (Table S1) for further analysis. Enriched GO terms among the differentially expressed genes were identified using DAVID (david.ncifcrf.gov).

#### CUT&Tag mapping and peak calling

Adaptor sequences in CUT&Tag paired-end reads were trimmed using Trim Galore! (Galaxy Tool Shed) and the trimmed reads were mapped to the *Drosophila melanogaster* (dm6) genome assembly using Bowtie2 (Langmead and Salzberg, 2012). bigWig coverage tracks were generated by normalization to the effective genome size (dm6). Peak calling of the CUT&Tag reads was performed using MACS2 (Feng et al., 2012), and overlapped peaks between different replicates/antibodies were identified using the Join tool (Galaxy Tool Shed). The obtained peaks were annotated with ChIPseeker.

## Supplementary Material

10.1242/develop.201548_sup1 Supplementary information
