# Supplementary material for: Separation of transcriptional repressor and activator functions in Drosophila HDAC3
Source: Development. 2023 Aug 2;150(15):dev201548. doi: 10.1242/dev.201548 (PMC10445730; doi:10.1242/dev.201548)
Supplement: Supplementary information [file develop-150-201548-s1.pdf]

A K26A mutant

```
                                               K26A
HDAC3_Dmel    -----MTDRRVSYFYNADVGNFHYGAGHPMKPQRLAVTHSLVMNYGLHKKMKIYRPYKAS   55
HDAC3_Human   ------MAKTVAYFYDPDVGNFHYGAGHPMKPHRLALTHSLVLHYGLYKKMIVFKPYQAS   54
HDAC1_Human   MAQTQGTRRKVCYYYDGDVGNYYYGQGHPMKPHRIRMTHNLLLNYGLYRKMEIYRPHKAN   60
HDAC1_Dmel    --MQSHSKKRVCYYYDSDIGNYYYGQGHPMKPHRIRMTHNLLLNYGLYRKMEIYRPHKAT   58
```

B Y303F mutant

```
                     Y303F
HDAC3_Dmel    LVVGGGGYTLRNVARCWTHETSLLVDQDIENDLPATEYYDFFAPDFTLHPEINSRQDNAN   355
HDAC3_Human   LVLGGGGYTVRNVARCWTYETSLLVEEAISEELPYSEYFEYFAPDFTLHPDVSTRIENQN   350
HDAC1_Human   LMLGGGGYTIRNVARCWTYETAVALDTEIPNELPYNDYFEYFGPDFKLHISP-SNMTNQN   354
HDAC1_Dmel    LMVGGGGYTIRNVSRCWTYETSVALAVEIANELPYNDYFEYFGPDFKLHISP-SNMTNQN   352
```

C HEBI mutant

```
                                                                K47E
HDAC3_Dmel    -----MTDRRVSYFYNADVGNFHYGAGHPMKPQRLAVTHSLVMNYGLHKKMKIYRPYKAS   55
HDAC3_Human   ------MAKTVAYFYDPDVGNFHYGAGHPMKPHRLALTHSLVLHYGLYKKMIVFKPYQAS   54
HDAC1_Human   MAQTQGTRRKVCYYYDGDVGNYYYGQGHPMKPHRIRMTHNLLLNYGLYRKMEIYRPHKAN   60
HDAC1_Dmel    --MQSHSKKRVCYYYDSDIGNYYYGQGHPMKPHRIRMTHNLLLNYGLYRKMEIYRPHKAT   58

                  LR60TK
HDAC3_Dmel    AQDMLRFHSDEYIAYLQQVTPQNIQCNSVAYTKYLAHFSVGEDCPVFDGLFDFCAMYTGA   115
HDAC3_Human   QHDMCRFHSEDYIDFLQRVSPTNMQ----GFTKSLNAFNVGDDCPVFPGLFEFCSRYTGA   110
HDAC1_Human   AEEMTKYHSDDYIKFLRSIRPDNMS----EYSKQMQRFNVGEDCPVFDGLFEFCQLSTGG   116
HDAC1_Dmel    ADEMTKFHSDEYVRFLRSIRPDNMS----EYNKQMQRFNVGEDCPVFDGLYEFCQLSAGG   114

        EGAQ118AAAV  HNH125KQA
HDAC3_Dmel    SLEGAQKLNHNHSDICINWSGGLHHAKKFEASGFCYVNDIVIGILELLKYHPRVLYIDID   175
HDAC3_Human   SLQGATQLNNKICDIAINWAGGLHHAKKFEASGFCYVNDIVIGILELLKYHPRVLYIDID   170
HDAC1_Human   SVASAVKLNKQQTDIAVNWAGGLHHAKKSEASGFCYVNDIVLAILELLKYHQRVLYIDID   176
HDAC1_Dmel    SVAAAVKLNKQASEICINWGGGLHHAKKSEASGFCYVNDIVLGILELLKYHQRVLYIDID   174
```

D

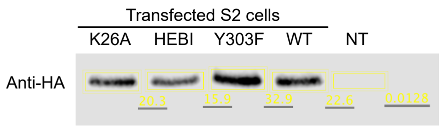

Fig. S1. Sequence alignment of *Drosophila melanogaster* and human HDAC3 and HDAC1 proteins. A-C) *Drosophila melanogaster* HDAC3 mutations expected to interfere with catalytic activity were modelled on human HDAC3. Red rectangles indicate the amino acid substitutions in each mutant. **A)** K26A **B)** Y303F **C)** HEBI. **D)** Western-blot shows protein extracts from S2 cells transfected wild-type and mutant HA-tagged HDAC3 after immunoprecipitation with an anti-HA antibody.

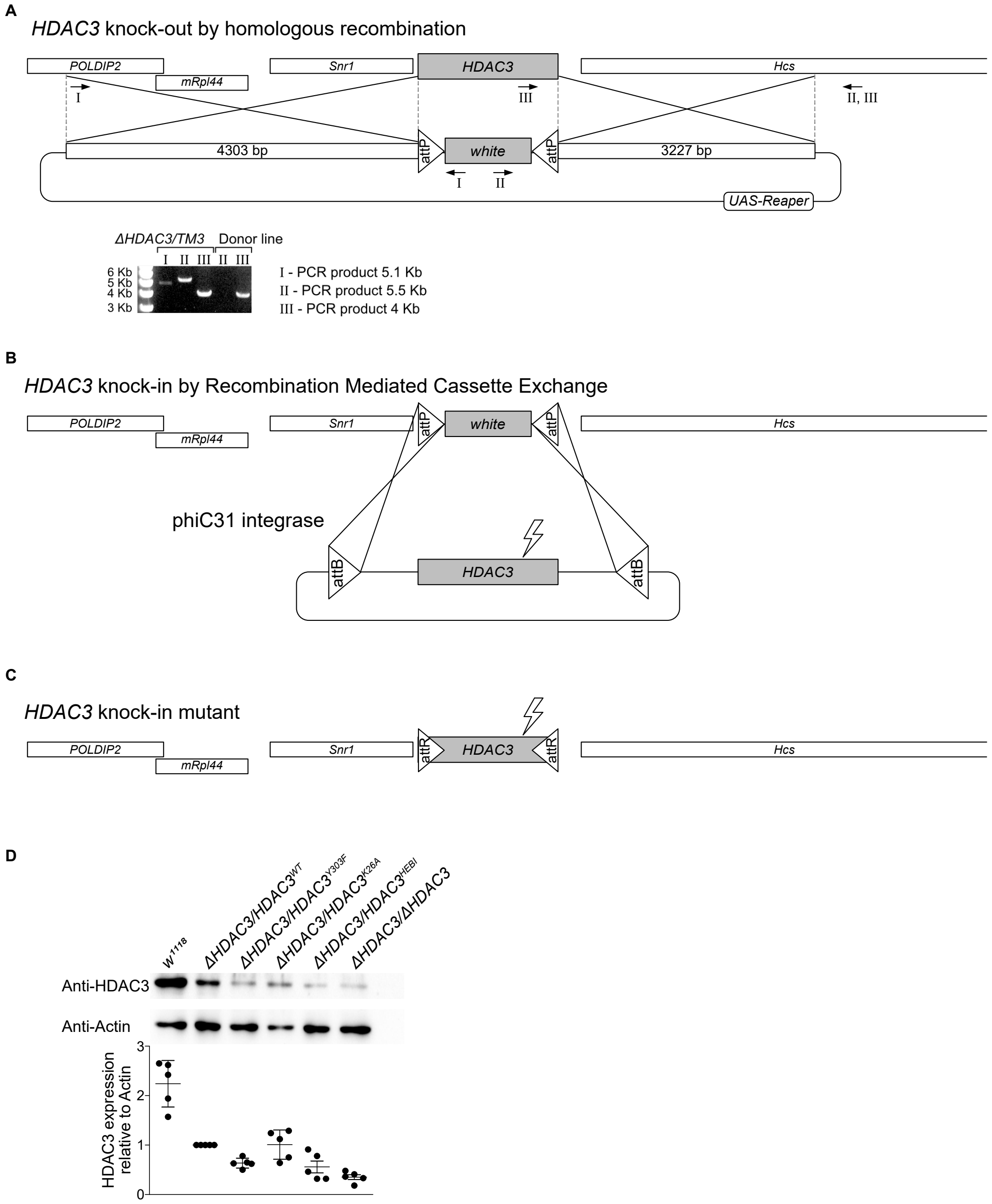

Fig. S2. Generation of HDAC3 knock-in mutant flies. A) The HDAC3 knock-out was created by replacement of the HDAC3 transcription unit with a *mini-white* (*white*) gene flanked by attP sites by homologous recombination. I to III indicate the locations of PCR primer pairs used to confirm the recombination in the balanced ΔHDAC3 line (ΔHDAC3/TM3) and compared to the donor line before recombination. **B, C)** The HDAC3 wild-type or mutant sequences were re-introduced into the endogenous HDAC3 locus using a recombination-mediated cassette exchange (RMCE)-based strategy with the phiC31 integrase. **D)** Western-blot from second instar larvae showing the levels of HDAC3 relative to Actin in wild-type (w1118) and trans-heterozygous HDAC3 mutant animals.

Development • Supplementary information

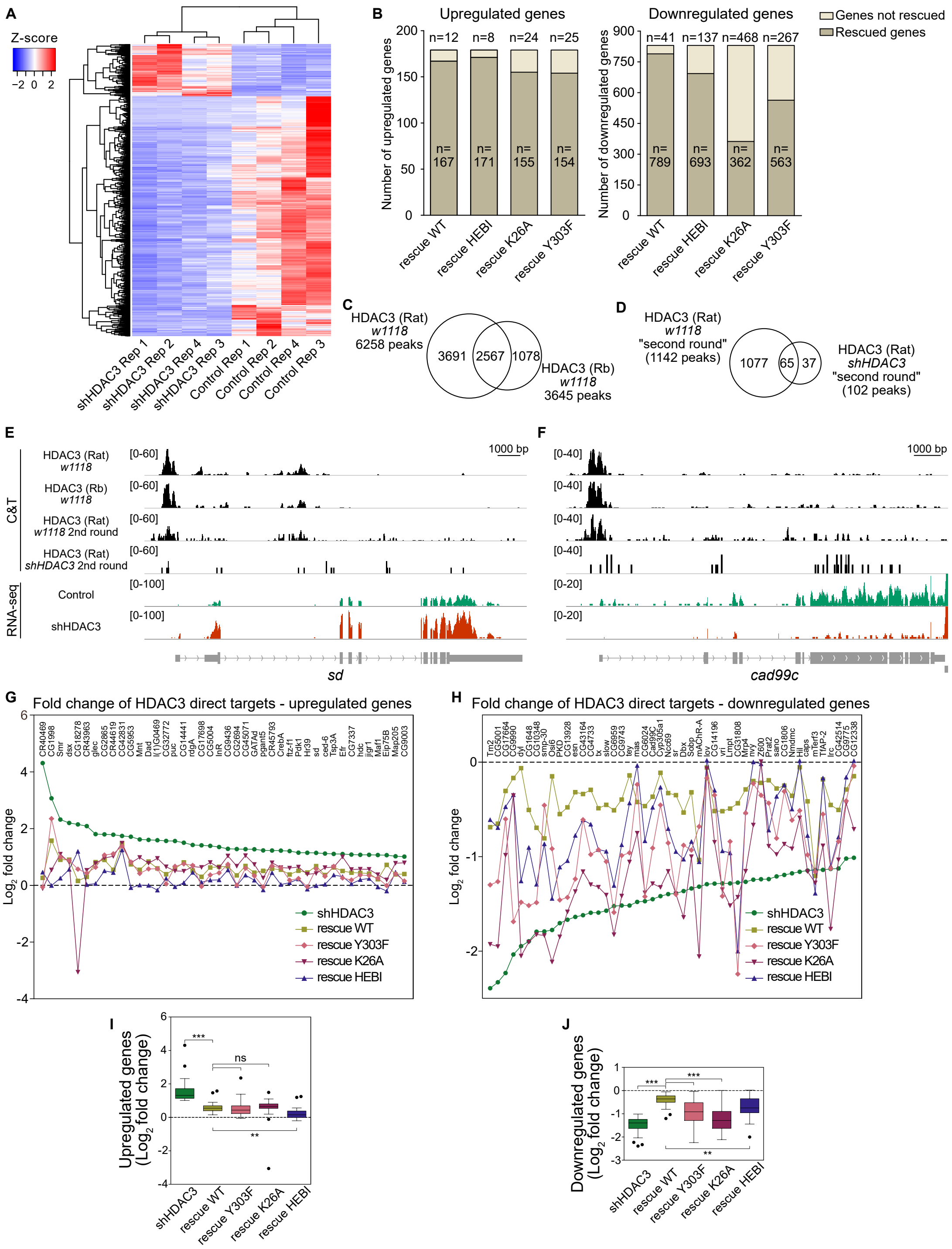

Fig. S3. RNA-seq and CUT&Tag in control and HDAC3 shRNA knock-down embryos. A) Heatmap showing normalized expression in HDAC3 shRNA and Control replicates standardized to Z-score by subtracting the mean and dividing by the standard deviation of the significantly changed genes (log_2 fold change ≥1; FDR <0.05). **B)** Number of differentially expressed genes between HDAC3 shRNA and

Development • Supplementary information

Control ($\log_2$ fold change ≥1, FDR ≤0.05) and the number of genes rescued by the wild-type or mutant HDAC3 shRNA-resistant rescue transgenes. **C)** Number of total and overlapped peaks between HDAC3 CUT&Tag data generated with two different antibodies (Rat and Rabbit) in 2-4h old w^{1118} embryos. **D)** Number of total and overlapped peaks between HDAC3 CUT&Tag in 2-4h old w^{1118} and HDAC3 shRNA embryos. **E, F)** Genome browser screenshots of one up-regulated (*sd*) and one down-regulated gene (*cad99c*) with tracks for HDAC3 CUT&Tag with the two different antibodies (Rat and Rabbit) in 2-4h old w^{1118} embryos, the second CUT&Tag experiment in 2-4h old w^{1118} and HDAC3 shRNA embryos and for RNA-seq signal (HDAC3 shRNA and Control). **G, H)** Expression fold change of individual HDAC3 direct targets in HDAC3 shRNA and in embryos rescued by wild-type or mutant HDAC3 relative to Control. The dashed line intersecting the 0 on the Y axis represents the Control (Tub-Gal4). **I, J)** Box-plots showing the average fold expression change of HDAC3 direct target genes in HDAC3 shRNA and in embryos rescued by wild-type or mutant HDAC3 relative to Control.. The dashed line intersecting the 0 on the Y axis represents the Control (Tub-Gal4/+). A two-tailed, unpaired Student's t test was applied to compare different genotypes and error bars show standard deviation.

Development • Supplementary information

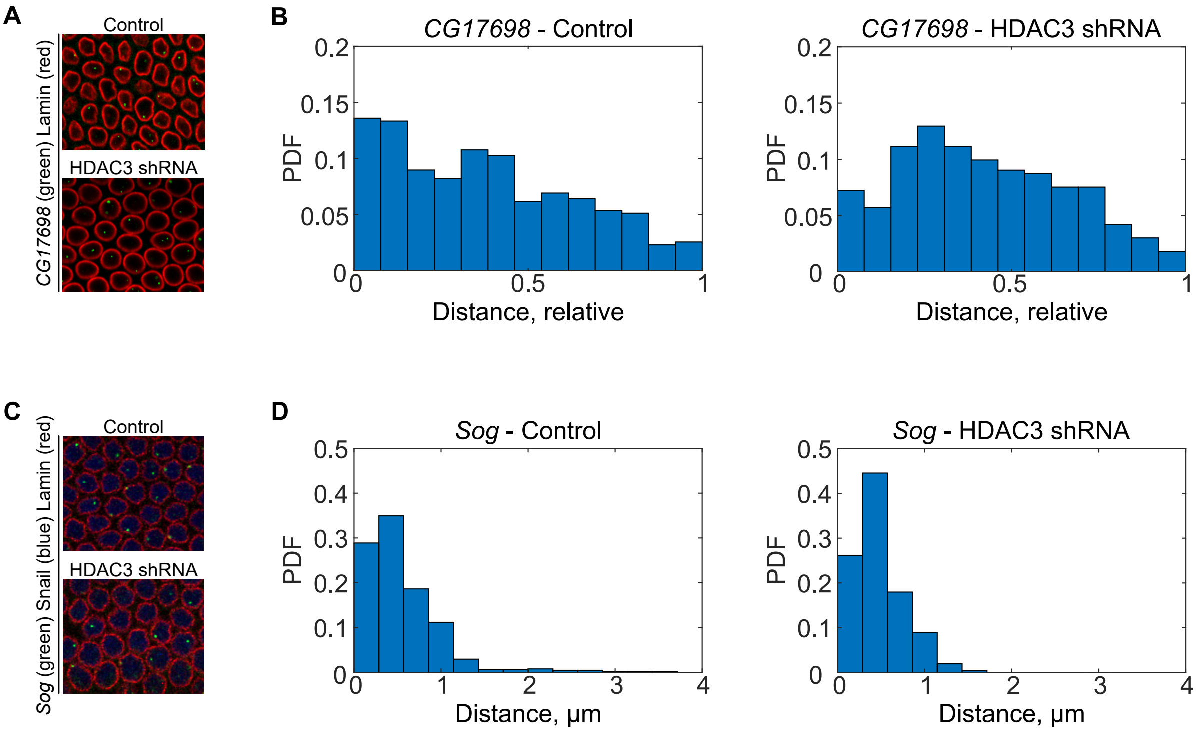

Fig. S4. *CG17698* is repressed by nuclear lamina targeting, whereas the *sog* gene is not. A) Image of DNA FISH in 2-4h old wild-type (Control) and HDAC3 shRNA embryos using a *CG17698* probe (green) followed by an immunostaining for Lamin (red) and DAPI staining (blue). **B)** Histograms of the relative distance of the *CG17698* locus to the nuclear periphery (lamin) based on z-projections, i.e., 2D distance, in Control and in HDAC3 shRNA embryos. X-axis: 0 = lamin and 1 = center of the nucleus., Y-axis: the probability density function, i.e. the number or counts per bin normalized by the total number of reads. **C)** Image of DNA FISH in 2-4h old wild-type (Control) and HDAC3 shRNA embryos using a probe *sog* (green) followed by an immunostaining for Lamin (red) and Snail (blue). Snail staining indicates nuclei in the mesoderm. **D)** Measurement of the distance of the *sog* locus to the nuclear periphery in Control and in HDAC3 shRNA embryos.

Development • Supplementary information

Table S1. Differentially expressed genes in HDAC3 knock-down and rescued embryos. RNA-seq from HDAC3 knock-down and rescued embryos showing up- and down-regulated genes from DESeq2 analysis, if they are maternal and/or zygotic, as well as HDAC3-bound up- and down-regulated genes.

Click here to download Table S1

Table S2. Gene ontology analysis of up- and down-regulated genes in HDAC3 knock-down embryos.

Click here to download Table S2

Table S3. List of primers.

Primer name	Primer sequence (5' - 3')	Notes
**HDAC3 knock in constructs**		
Hdac3LAfor	TCCCCAACGGCTGCTACC	
Hdac3LAAcc65Irev	GGTACCAGCGTAAGCAGTAAAGAGTATCA	
Hdac3RAAscIfor	GGCGCGCCTAGTGTACTAAAAATGGA	
Hdac3RAAgeIrev	TACCGGTGGTTTGCGAGAGTGCGAC	
pABCHDAC3frw	GAGAAAGCTTCGCCGCTTTAAGTTGTCTTC	
pABCHDAC3rev	GAGAAAGCTTACAAAATAAGCTGATACTCTTTACTGC	
HDAC3m knockin test F	GGACAAGCTGGTTGATGCTA	
HDAC3m knockin test R	AGGCCCCTCTATCAACGATT	
**HDAC3 knock down resistant transgenes**		
HDAC3GR F	acacta**TCTAGA**GGGCCGCTGTAGTGGCTCTATG	
HDAC3GR R	atacaA**TCTAG**AATGCGGCGCAGAAGGAGTG	
HDAC3res F	TACTGTAG AATGTAGAATAAGGATTCGA	
HDAC3res R	TTAGCTCTCC GCCGAATCGGGCTTGTCTT	
HDAC3 Y303F F	TTCACCTTACGGAACGTGGC	Y303F TAC>TTC
HDAC3 Y303F R	GCCACCGCCTCCGACC	
HDAC3 K26A F	GCGCCGCAACGCCTGGCG	
HDAC3 K26A R	CATGGGATGCCCGGCTCCATAG	
HDAC3 K47E F	gaGATCTACAGGCCGTACAAGTGAG	K47E AAG>GAG
HDAC3 K47E R	CATTTTCTTGTGCAGTCCGTAG	
HDAC3 HIm F	CTGAACAAGCAAGCCAGCGACATATGCATAAACTGGTCGG	HNH125-127KQA
HDAC3 HIm R	CTTCACGGCGGCGGCCAGCGAGGCACCCGTGTAC	EGAQ118-121AAAV
HDAC3 Em F	AAGTTCCACAGCGACGAGTACATC	R61K CGC > AAG
HDAC3 Em R	ggtCATGTCCTGCGCACTGG	L60T CTC > ACC
**HA-tagged HDAC3**		
HDAC3 C Hind III F	AAGCTTACTTTCTGCCGAATCGGGCTT	
HDAC3 C Hind III R	TAGTATTGGATAATGTAGAATAAGGATTCGA	
HDAC3 C tag F	AGACAAGCCCGATTCGGCAGAAAGTCGATCCTACCCCTACGATGTT	
HDAC3 C tag R	CCTTATTCTACATTATCCAATACTACTTGTCATCATCGTCCTTGTAGTCGAG	
**Smr constructs**		
Smr full length F	CCAGCACAGTGGCGGCCGCTCGAGTATGGAAGCAGCTATACGCTCCA	Exon 1
smr 9R-2	GGCTTACCTTCGAAGGGCCCTCTAGACTGTGAATGGATGCTGCATTTG	
smr 9F-2	CATCAAATGCAGCATCCATTCACAGCCCCCACTGGTCTTACAG	Exon 2
smr 10R-2	GGCTTACCTTCGAAGGGCCCTCTAGACTGAGGATGATAGGCCTC	
smr 10F-2	CAGCACCGAGGCCTATCATCCTCAGGTGGAGGCCATTTCGCCG	Exon 3-5
smr 11R-2	GGCTTACCTTCGAAGGGCCCTCTAGACTTTGCCATCGAGGGCGAG	
smr 11F-2	GAGATTCTCGCCCTCGATGGCAAAGACAAATTGGCCAGCTGCTTTG	Exon 6
smr 12R-2	GGCTTACCTTCGAAGGGCCCTCTAGACTATTAACATGATCGGCTGGC	
smr 12F-2	CATCGCCAGCCGATCATGTTAATAGCACTCCGAGTCCGCATCG	Exon 7-9
Smr full length R-2	GGCTTACCTTCGAAGGGCCCTCTAGAATCTTCGTCGCTGAGTGCAT	
smrDAD F	CCAGCACAGTGGCGGCCGCTCGAGTATGAATGTTGCCGCCGGATTGGGTA	
smrDAD R	GGCTTACCTTCGAAGGGCCCTCTAGAATCCATGATCTCCTCGAGATCCGCT	
**DNA FISH**		
sog fish f1	TGCCCCATGTGCGACTATTA	1666bp
sog fish r1	TAAGAAAGAGCGGTCCAGGG	
sog fish f2	GGGTAGCTGATGGGAATGGT	1225bp
sog fish r2	GACCATAAAGCGGCACCCAA	
sog fish f3	TCTACCTGCGATTACGGGGA	1516bp
sog fish r3	GATTGCACATCGGTCGTTCG	
sog fish f4	GGGTCGACAGTCCGAGAATC	1368bp
sog fish r4	TGGTAGAGCCGGCTGAGATA	
sog fish f5	AAATGTTTTGGCCTCACGGC	1346bp
sog fish r5	CACACACCGGCTACTGTTCT	
sog fish f6	ACTGGTCCGCATCGAATGTT	1297bp
sog fish r6	CTACACAGCGATTTGTCGCC	
CG32772 fish f1	GCGTAACATAGCGTTTGGTGCTGTC	1214bp
CG32772 fish r1	ATATGGCCACAATCAGCATGGAGGG	
CG32772 fish f2	AGCATGGTGGAGTTCGCCTTTGTTA	1666bp
CG32772 fish r2	CTTTGTTGCTGACTTTTGCCCCCTC	
CG32772 fish f3	AATGACAGAAATTGCGCCAAGGCAG	1475bp
CG32772 fish r3	TGTGAGCGGGGGAGTAGTAGTGAAA	
CG32772 fish f4	GCACGGGAATTTGCAATCCGTTCTT	1450bp
CG32772 fish r4	TTGATCTCGATCTTGATGGCCGTGG	
CG32772 fish f5	ATCGGCGAGTGGACTTCCAAAACAT	1314bp
CG32772 fish r5	TCGTTTCTCTCTCCCCCAGGACATT	
CG32772 fish f6	TGCCAATGAACAGCGGATACGAACT	1585bp
CG32772 fish r6	CTCTTCAACTACGGGCTGCATCACA	
CG17698 fish f1	GCCATCTGTATATTGGCGCA	1340bp
CG17698 fish r1	TCGTTCGCACTCCCACTAAC	
CG17698 fish f2	TGGTCTTTAGGGCGGATTGT	1379bp
CG17698 fish r2	GGGCTATACGGTACATTCGGG	
CG17698 fish f3	AACCAGCTTTTCGGGAGTCT	1394bp
CG17698 fish r3	TTCGCAACGAGATGTGAGCA	
CG17698 fish f4	ATTGCGTTTGGGTATTGGAG	1113bp
CG17698 fish r4	AACATAACGCTCACAAAGCAA	
CG17698 fish f5	GCGTCAAGCTGGTCTTATGC	1293bp
CG17698 fish r5	AACAGTGTGTTGGGATGACGA	

Development • Supplementary information
