# Supplementary material for: Separation of transcriptional repressor and activator functions in Drosophila HDAC3
Source: Development. 2023 Aug 2;150(15):dev201548. doi: 10.1242/dev.201548 (PMC10445730; doi:10.1242/dev.201548)
Supplement: Supplementary file 1 [file develop-150-201548_review_history.pdf]

Separation of transcriptional repressor and activator functions in *Drosophila* HDAC3

Min Tang, Isabel Regadas, Sergey Belikov, Olga Shilkova, Lei Xu, Erik Wernersson, Xuewen Liu, Hongmei Wu, Magda Bienko and Mattias Mannervik
DOI: 10.1242/dev.201548

Editor: Thomas Lecuit

Review timeline

Original submission:	17 December 2022
Editorial decision:	9 February 2023
First revision received:	13 June 2023
Accepted:	11 July 2023

Original submission

First decision letter

MS ID#: DEVELOP/2022/201548

MS TITLE: Separation of transcriptional repressor and activator functions in Drosophila HDAC3

AUTHORS: Min Tang, Isabel Regadas, Sergey Belikov, Olga Shilkova, Lei Xu, Erik Wernersson, Xuewen Liu, Hongmei Wu, Magda Bienko, and Mattias Mannervik

I apologise for the long delay in getting back to you with a decision. I have now received all the referees reports on the above manuscript, and have reached a decision. The referees' comments are appended below, or you can access them online: please go to BenchPress and click on the 'Manuscripts with Decisions' queue in the Author Area.

The overall evaluation is positive and we would like to publish a revised manuscript in Development, provided that the referees' comments can be satisfactorily addressed. Please attend to all of the reviewers' comments in your revised manuscript and detail them in your point-by-point response. If you do not agree with any of their criticisms or suggestions explain clearly why this is so. If it would be helpful, you are welcome to contact us to discuss your revision in greater detail. Please send us a point-by-point response indicating your plans for addressing the referee's comments, and we will look over this and provide further guidance.

Reviewer 1

Advance summary and potential significance to field

This manuscript addresses HDAC3 function in the Drosophila early embryo by investigating the effect of 3 mutations, initially studied in vertebrate HDAC3, which disrupt HDAC3Â's catalytic activity. The results suggest that HDAC3 has separable activation and repressive functions, and that repression, but not activation, is largely independent of catalysis. The authors suggest different mechanisms for non-enzymatic repression, including association with the nuclear periphery following H3K9me2 modification.

© 2023. Published by The Company of Biologists under the terms of the Creative Commons Attribution License (https://creativecommons.org/licenses/by/4.0/).

Comments for the author

Overall, I think that the data support the conclusions, but I found the manuscript a bit difficult to follow in places. I have the following minor comments that mostly relate to increasing the clarity for the reader:

For Table 1, are the data showing the percentage of mutant larvae relative to half the number of balanced larvae? It would help the reader to clarify this. The idea that the Y303F and K26A chromosomes have second site mutations is odd – why would additional mutations be introduced with cassette exchange?

Did the authors only find a single positive line for exchange with the Y303F or K26A cassettes and if not was there not a line that did not appear to have second site mutations?

In the Western blot in Fig S2D, are the larvae analysed from crosses of HDAC3D/balancer females x HDAC3 mutation of interest/balancer males? Based on the levels with K26A, the authors say 'This shows that the poor survival of K26A mutants cannot be explained by reduced protein expression, and therefore that the catalytic activity of HDAC3 is essential for viability'. However, this conclusion does not entirely make sense to me as 58% of the HDAC3/K26A embryos survive to larvae. Do they mean the catalytic activity is essential for full survival or have I missed something? Can the authors speculate about the mechanism by which the Y303F mutation allows upregulation of the silenced endogenous HDAC3 – do they think this is mediated at the transcriptional level or is it loss of silencing? If it is the latter, why might this be occurring only with the Y303F mutant protein?

Based on the rescue by HEBI but not K26A in the transgene system (line 196), are the authors concluding that K26A is required for something else other than SMRTER interaction?
As the wildtype HDAC3 transgene rescues the knockdown lethality quite poorly yet rescues >93% of the up-/down-regulated genes, do the authors favour the interpretation that one or more of the 53 genes not rescued in the early embryo is essential for viability or that the lethality is caused by accumulated gene expression defects?

The authors mention that the HDAC3 shRNA knock-down also affects maternally contributed embryonic transcripts – how many of the up or downregulated transcripts are due to changes in maternal mRNAs?

Are any of the candidate mRNAs up or downregulated in the RNA-seq from the Y303F mutant transgene rescue (Fig S3B) able to potentially explain why the segmentation phenotype is so prevalent for that specific mutation?

The authors conclude that ~10% of HDAC3-repressed genes could be regulated by H3K9me2 modification leading to nuclear lamina targetting, and mention this as a repression mechanism in the Abstract. However, the nuclear location of only one of the 4 H3K9me2 marked genes has been tested – are the other 3 genes also more lamina associated in wildtype embryos relative to HDAC3 knockdown?

<u>Reviewer 2</u>

Advance summary and potential significance to field

In this work, Tang and colleagues study Drosophila HDAC3 and, in particular, the role of its catalytic activity in its functions as a co-transcriptional activator and repressor. They take advantage of three mutants modeled on mammalian HDAC3, which either disrupt its catalytic activity or disrupt both its catalytic activity and its interaction with the SMRTER complex. Biochemical assays in S2 cells showed how the Y30F, the K26A, and the HEBI mutants lost their catalytic activities. In contrast, only the HEBI mutant (completely) and the K26A mutant (partially) also lost the interaction with the SMRTER complex. The in vitro experiments (Figure 1) clearly show that these mutations can be used to study HDAC3 function in vivo. I suggest a revision of the manuscript.

© 2023. Published by The Company of Biologists under the terms of the Creative Commons Attribution License (https://creativecommons.org/licenses/by/4.0/).

Major points:

1. Figure 2B. Several points need to be addressed in this dot plot showing hatching rates. In general more statistics should be performed and added to the plot.

1a) The hatching rate of tub-Gal4/+ (control) is too low (less than 50%) and also very variable for a control strain. Have the authors assessed the hatching rate of a wild-type strain? Unfortunately, the low hatching rate of the control affects the results of the hatching rates of all the other genotypes. Could this be due to the low HDAC3 protein levels in the rescues, as shown in Figure 2C?

-The wild-type rescue of HDAC3-KD shows a modest increase from HDAC3-KD hatching rate levels. Unfortunately, no statistics are shown to compare HDAC3-KD to wild-type rescue. Such a slight difference between KD and wild-type rescue hatching rate levels might cause problems interpreting the result. We suggest repeating the experiments while trying to determine the cause of the low rescue.

- Y303F rescue hatching rate levels are similar to wild-type rescue levels. However, in figure 2D, Y303F rescue shows higher expression levels of endogenous WT HDAC3 mRNA. Can the authors try to speculate what is happening and try to find a solution?

- K26A shows better results, which could be used for further analyses. Have the authors considered using this line instead of Y303F?

- HEBI rescue hatching rate levels are higher than wild-type rescue. Can the authors try to interpret and explain this result?

2. Figure 3. As mentioned in the comments for Figure 2, the authors should incorporate statistics and mention the number of biological replicates in the figure legend. Referring to the Y303F line and the high expression of WT HDAC3 mRNA, as mentioned in the previous comment, the authors would need to clarify this or consider removing this data.

3. Figure 4. It is reassuring that the authors observe that the vast majority of the genes up or down-regulated in HDAC3-KD are efficiently rescued by the wild-type construct (Figure 4B). Given such a high rescue in gene expression, despite suboptimal HDAC3 protein levels and phenotype rescue, could this difference be due to non-transcriptional roles of HDAC3?

4. Figure 5. Given the slight overlap between HDAC3 targets and the H3K9me2 or the H3K27me3 targets, it is hard to show an interplay between HDAC3 and Polycomb-repression or H3K9me2/Lamina-repression. I would suggest the authors to measure also other targets as in Figure 5E.

Comments for the author

In this work, Tang and colleagues study Drosophila HDAC3 and, in particular, the role of its catalytic activity in its functions as a co-transcriptional activator and repressor. They take advantage of three mutants modeled on mammalian HDAC3, which either disrupt its catalytic activity or disrupt both its catalytic activity and its interaction with the SMRTER complex. Biochemical assays in S2 cells showed how the Y30F, the K26A, and the HEBI mutants lost their catalytic activities. In contrast, only the HEBI mutant (completely) and the K26A mutant (partially) also lost the interaction with the SMRTER complex. The in vitro experiments (Figure 1) clearly show that these mutations can be used to study HDAC3 function in vivo. I suggest a revision of the manuscript.

Major points:

1. Figure 2B. Several points need to be addressed in this dot plot showing hatching rates. In general more statistics should be performed and added to the plot.

© 2023. Published by The Company of Biologists under the terms of the Creative Commons Attribution License (https://creativecommons.org/licenses/by/4.0/).

1a) The hatching rate of tub-Gal4/+ (control) is too low (less than 50%) and also very variable for a control strain. Have the authors assessed the hatching rate of a wild-type strain? Unfortunately, the low hatching rate of the control affects the results of the hatching rates of all the other genotypes. Could this be due to the low HDAC3 protein levels in the rescues, as shown in Figure 2C?

-The wild-type rescue of HDAC3-KD shows a modest increase from HDAC3-KD hatching rate levels. Unfortunately, no statistics are shown to compare HDAC3-KD to wild-type rescue. Such a slight difference between KD and wild-type rescue hatching rate levels might cause problems interpreting the result. We suggest repeating the experiments while trying to determine the cause of the low rescue.

- Y303F rescue hatching rate levels are similar to wild-type rescue levels. However, in figure 2D, Y303F rescue shows higher expression levels of endogenous WT HDAC3 mRNA. Can the authors try to speculate what is happening and try to find a solution?

- K26A shows better results, which could be used for further analyses. Have the authors considered using this line instead of Y303F?

- HEBI rescue hatching rate levels are higher than wild-type rescue. Can the authors try to interpret and explain this result?

2. Figure 3. As mentioned in the comments for Figure 2, the authors should incorporate statistics and mention the number of biological replicates in the figure legend. Referring to the Y303F line and the high expression of WT HDAC3 mRNA, as mentioned in the previous comment, the authors would need to clarify this or consider removing this data.

3. Figure 4. It is reassuring that the authors observe that the vast majority of the genes up or down-regulated in HDAC3-KD are efficiently rescued by the wild-type construct (Figure 4B). Given such a high rescue in gene expression, despite suboptimal HDAC3 protein levels and phenotype rescue, could this difference be due to non-transcriptional roles of HDAC3?

4. Figure 5. Given the slight overlap between HDAC3 targets and the H3K9me2 or the H3K27me3 targets, it is hard to show an interplay between HDAC3 and Polycomb-repression or H3K9me2/Lamina-repression. I would suggest the authors to measure also other targets as in Figure 5E.

First revision

Author response to reviewers' comments

We thank the reviewers for their appreciation of our work and for their useful comments and suggestions. We are happy that the reviewers find that “the data support the conclusions“, and that our experiments “clearly show that these mutations can be used to study HDAC3 function in vivo”.

The reviewers also noticed that the manuscript could benefit from further support for nuclear lamina targeting as a repression mechanism. Based on the reviewers´ critiques we have performed new experiments. In the revised manuscript, we have added additional support to the study in two main areas. We provide 1) further evidence for HDAC3-mediated nuclear lamina targeting by additional FISH experiments. 2) We demonstrate a clear, but not full, rescue with the wt HDAC3 transgene by repeating the embryo survival assays.

Our response to all of the comments is found below.

Reviewer 1 Advance Summary and Potential Significance to Field:
This manuscript addresses HDAC3 function in the Drosophila early embryo by investigating the effect of 3 mutations, initially studied in vertebrate HDAC3, which disrupt HDAC3’s catalytic

© 2023. Published by The Company of Biologists under the terms of the Creative Commons Attribution License (https://creativecommons.org/licenses/by/4.0/).

activity. The results suggest that HDAC3 has separable activation and repressive functions, and that repression, but not activation, is largely independent of catalysis. The authors suggest different mechanisms for non- enzymatic repression, including association with the nuclear periphery following H3K9me2 modification.

Reviewer 1 Comments for the Author:
Overall, I think that the data support the conclusions, but I found the manuscript a bit difficult to follow in places. I have the following minor comments that mostly relate to increasing the clarity for the reader:

Response: We thank the reviewer for a careful reading and for pointing out unclear parts of the manuscript.

For Table 1, are the data showing the percentage of mutant larvae relative to half the number of balanced larvae? It would help the reader to clarify this. The idea that the Y303F and K26A chromosomes have second site mutations is odd - why would additional mutations be introduced with cassette exchange?

Response: The data show the percent of 1st instar larvae lacking a balancer chromosome (YFP-) that reach the 3rd instar stage. This has been clarified in the legend to Table 1.
It is unexpected that cassette exchange introduces second site mutations, but we have observed this also for other genes.

Did the authors only find a single positive line for exchange with the Y303F or K26A cassettes and if not, was there not a line that did not appear to have second site mutations?

Response: Unfortunately, we have only kept one line for each mutant and realized the issue with second site mutations afterwards.

In the Western blot in Fig S2D, are the larvae analysed from crosses of HDAC3D/balancer females x HDAC3 mutation of interest/balancer males? Based on the levels with K26A, the authors say ‘This shows that the poor survival of K26A mutants cannot be explained by reduced protein expression, and therefore that the catalytic activity of HDAC3 is essential for viability’. However, this conclusion does not entirely make sense to me as 58% of the HDAC3/K26A embryos survive to larvae. Do they mean the catalytic activity is essential for full survival or have I missed something?

Response: Yes, the larvae are from HDAC3delta/balancer females x HDAC3 point mutant/balancer males. Although 58% of K26A 1st instar larvae make it to the 3rd instar, none eclose as adult flies. We have clarified this on p. 7 line 150 and in the legend to Table 1.

Can the authors speculate about the mechanism by which the Y303F mutation allows upregulation of the silenced endogenous HDAC3 - do they think this is mediated at the transcriptional level or is it loss of silencing? If it is the latter, why might this be occurring only with the Y303F mutant protein?

Response: We have no good explanation for why the Y303F mutant upregulates endogenous HDAC3 expression, but speculate that it has to do with genetic compensation (PMID: 28704371), which is poorly understood, but may involve epigenetic remodeling (PMID: 30944477). We have added a sentence about this speculation on p. 9, line 193. Why this only occurs in the Y303F mutant is a mystery to us.

Based on the rescue by HEBI but not K26A in the transgene system (line 196), are the authors concluding that K26A is required for something else other than SMRTER interaction?

Response: It is possible that K26A is required for something other than the interaction with SMRTER since this mutation disrupts the HDAC3 activator function but is able to repress to the same extent as the wild-type protein. Alternatively, repression is independent of SMRTER in this context, and HEBI may instead have gained a function that allows it to repress transcription better than wild-type, which allows it to rescue the hatching phenotype.

© 2023. Published by The Company of Biologists under the terms of the Creative Commons Attribution License (https://creativecommons.org/licenses/by/4.0/).

As the wildtype HDAC3 transgene rescues the knockdown lethality quite poorly yet rescues >93% of the up-/down-regulated genes, do the authors favour the interpretation that one or more of the 53 genes not rescued in the early embryo is essential for viability or that the lethality is caused by accumulated gene expression defects?

Response: The amount of gene expression rescue depends on the cut-off used. Here it is defined as a fold-change difference less than 2 between wt HDAC3 shRNA-resistant and control tub-Gal4/+ embryos. We have clarified this on p. 11, line 255.

As can be seen in Fig. 4H-K, the wild-type transgene does not completely rescue expression of almost any gene to the level found in the control, most likely due to its poor expression, although it brings expression back to less than 2-fold (log2 1 or -1) versus the control. We therefore favour the idea that lethality is caused by an accumulated gene expression defect.

The authors mention that the HDAC3 shRNA knock-down also affects maternally contributed embryonic transcripts – how many of the up or downregulated transcripts are due to changes in maternal mRNAs?

Response: This is difficult to answer because many of our target genes are lowly expressed in the embryo, and therefore not present in lists of maternal/zygotic transcripts from other sources. We have compared our data to Ibarra-Morales et al. (PMID: 34853314), and of the 179 up-regulated genes, 64 are strictly maternal, 62 are maternal+zygotic, 8 are strictly zygotic and 45 not detected in the other dataset. Of the 830 down-regulated genes, 630 are not detected in other datasets, 18 purely maternal, 21 maternal+zygotic and 161 strictly zygotic. We have also looked in other publications, but most of the genes that are not detected are also missing from these data. Because of this, we have added information on maternal/zygotic where data is available to Table S1 (mentioned on p. 12, line 261) without referring to numbers in the text.

Are any of the candidate mRNAs up or downregulated in the RNA-seq from the Y303F mutant transgene rescue (Fig S3B) able to potentially explain why the segmentation phenotype is so prevalent for that specific mutation?

Response: We have looked for candidate genes that could explain the segmentation defects in this mutant, but have not found an obvious candidate. The uniquely mis-regulated genes in Y303F embryos are listed in Table 1 for reviewer, at the end of this document.

The authors conclude that ~10% of HDAC3-repressed genes could be regulated by H3K9me2 modification leading to nuclear lamina targetting, and mention this as a repression mechanism in the Abstract. However, the nuclear location of only one of the 4 H3K9me2 marked genes has been tested – are the other 3 genes also more lamina associated in wildtype embryos relative to HDAC3 knockdown?

Response: We have performed new FISH experiments for the 3 remaining H3K9me2-marked genes. Unfortunately, the probe failed for one of them and the quality of another experiment did not allow us to accurately quantify the distance to the nuclear periphery, but we did obtain data for one more locus. This locus, *CG17698*, is indeed more lamina-associated in wildtype embryos relative to HDAC3 knockdown, supporting our conclusion that one catalytic-independent repression mechanism used by HDAC3 is to help tether some loci to the nuclear periphery.

Reviewer 2 Advance Summary and Potential Significance to Field:
In this work, Tang and colleagues study Drosophila HDAC3 and, in particular, the role of its catalytic activity in its functions as a co-transcriptional activator and repressor. They take advantage of three mutants modeled on mammalian HDAC3, which either disrupt its catalytic activity or disrupt both its catalytic activity and its interaction with the SMRTER complex. Biochemical assays in S2 cells showed how the Y30F, the K26A, and the HEBI mutants lost their catalytic activities. In contrast, only the HEBI mutant (completely) and the K26A mutant (partially) also lost the interaction with the SMRTER complex.

The in vitro experiments (Figure 1) clearly show that these mutations can be used to study HDAC3

© 2023. Published by The Company of Biologists under the terms of the Creative Commons Attribution License (https://creativecommons.org/licenses/by/4.0/).

function in vivo. I suggest a revision of the manuscript.

Response: We thank the reviewer for a careful reading of the manuscript and for useful suggestions.

Major points:

1.Figure 2B. Several points need to be addressed in this dot plot showing hatching rates. In general, more statistics should be performed and added to the plot.

1a) The hatching rate of tub-Gal4/+ (control) is too low (less than 50%) and also very variable for a control strain. Have the authors assessed the hatching rate of a wild-type strain? Unfortunately, the low hatching rate of the control affects the results of the hatching rates of all the other genotypes. Could this be due to the low HDAC3 protein levels in the rescues, as shown in Figure 2C?

Response: We agree that this data was not that convincing. In order to be comprehensive, we plotted all of our survival assays in Fig. 2B. However, because of dry juice plates and other environmental circumstances, survival was very low for all genotypes in some cases. We have repeated the experiment and replaced this plot with a new Fig. 2B (5 biological replicates). Survival for the tub-Gal4/+ control strain is now around 75%.

We have also performed survival assays from a wild-type strain, results shown in Fig. 1 for Reviewer below. This shows that hatching rates are slightly (but not statistically significant) higher in w^{1118} than in the tub-Gal4/+ strain.

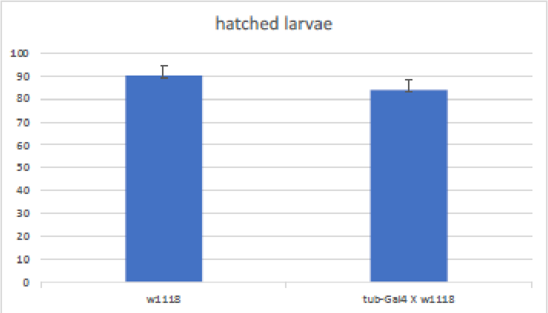

Figure 1 for Reviewer. Hatching rates for wild-type (w1118) and tub-Gal4/+ strains.
-The wild-type rescue of HDAC3-KD shows a modest increase from HDAC3-KD hatching rate levels. Unfortunately, no statistics are shown to compare HDAC3-KD to wild-type rescue. Such a slight difference between KD and wild-type rescue hatching rate levels might cause problems interpreting the result. We suggest repeating the experiments while trying to determine the cause of the low rescue.

Response: The wild-type rescue is more evident in the new experiments presented in new Fig. 2B, and statistics showing the difference between HDAC3-KD and rescue is now shown. We believe that the absence of full rescue is due to poor expression of the wt HDAC3 transgene. Some regulatory regions may be missing from the transgene (although all intergenic sequences between the flanking genes are included, see Fig. 2A), and the transgene is only present as one copy compared to the two endogenous alleles.

- Y303F rescue hatching rate levels are similar to wild-type rescue levels. However, in figure 2D, Y303F rescue shows higher expression levels of endogenous WT HDAC3 mRNA. Can the authors try to speculate what is happening and try to find a solution?

Response: As explained to Reviewer 1, we do not understand why endogenous wt HDAC3 is expressed in Y303F embryos, but speculate that it has to do with genetic compensation (PMID: 28704371).

- K26A shows better results, which could be used for further analyses. Have the authors considered

© 2023. Published by The Company of Biologists under the terms of the Creative Commons Attribution License (https://creativecommons.org/licenses/by/4.0/).

using this line instead of Y303F?

Response: We do focus our analyses on K26A instead of Y303F in Fig. 4.

- HEBI rescue hatching rate levels are higher than wild-type rescue. Can the authors try to interpret and explain this result?

Response: As shown in Fig. 4, the HEBI mutant rescues up-regulated genes better than wild-type, which we interpret to mean that it has improved repressor activity. We believe that this may explain why hatching rates are higher than wild-type. In the Discussion, we suggest that this may represent a function for HDAC3 outside of the SMRTER complex.

2. Figure 3. As mentioned in the comments for Figure 2, the authors should incorporate statistics and mention the number of biological replicates in the figure legend. Referring to the Y303F line and the high expression of WT HDAC3 mRNA, as mentioned in the previous comment, the authors would need to clarify this or consider removing this data.

Response: Statistics have now been included for Fig. 3. Given that Y303F produces a stronger segmentation defect than any other genotype, we prefer to maintain the data from this strain in Fig. 3. The presence of wild-type HDAC3 in these embryos is mentioned on line 234 and has been added to the Figure 3 legend.

3. Figure 4. It is reassuring that the authors observe that the vast majority of the genes up or down-regulated in HDAC3-KD are efficiently rescued by the wild-type construct (Figure 4B). Given such a high rescue in gene expression, despite suboptimal HDAC3 protein levels and phenotype rescue, could this difference be due to non-transcriptional roles of HDAC3?

Response: Although it is possible that HDAC3 has non-transcriptional roles, we favour an alternative explanation. As described in the response to Reviewer 1, the amount of gene expression rescue depends on the cut-off used. Here it is defined as a fold-change difference less than 2 between wt HDAC3 shRNA-resistant and control tub-Gal4/+ embryos. As can be seen in Fig. 4H-K, the wild-type transgene does not completely rescue expression of almost any gene to the level found in the control, most likely due to its poor expression.

4. Figure 5. Given the slight overlap between HDAC3 targets and the H3K9me2 or the H3K27me3 targets, it is hard to show an interplay between HDAC3 and Polycomb-repression or H3K9me2/Lamina- repression. I would suggest the authors to measure also other targets as in Figure 5E.

Response: We have now performed new FISH experiments and the results from *CG17698* is shown in new Fig. S4. FISH for two other targets failed, unfortunately. The new result gives further support to our conclusion that one out of several mechanisms by which HDAC3 represses transcription is through tethering of loci to the nuclear periphery.

Table 1 for reviewer. Genes uniquely mis-expressed in Y303F embryos.

Up (n=14) RpS5b CG32305
Gfrl rpr CG32301
CG31728
Cnx14D Cyp18a1
CG33500 bsh
CG30090
CR44498
CG9222
CG33160

Down (n=20)
CG5756
Ipod
CG3164

© 2023. Published by The Company of Biologists under the terms of the Creative Commons Attribution License (https://creativecommons.org/licenses/by/4.0/).

CG9503 haf
Phk-3
CG3902
CG42741
Cht2 slbo
CG11353
Ppn CG2781 mesh CG13615
CG9411
CG14245
Syn
CG42747
CG31036

Second decision letter

MS ID#: DEVELOP/2022/201548

MS TITLE: Separation of transcriptional repressor and activator functions in Drosophila HDAC3

AUTHORS: Min Tang, Isabel Regadas, Sergey Belikov, Olga Shilkova, Lei Xu, Erik Wernersson, Xuewen Liu, Hongmei Wu, Magda Bienko, and Mattias Mannervik
ARTICLE TYPE: Research Article

I am happy to tell you that your manuscript has been accepted for publication in Development, pending our standard ethics checks.

Reviewer 1

Advance summary and potential significance to field

See my original review.

Comments for the author

The authors have addressed all my concerns and I believe the paper is strengthened.

Reviewer 2

Advance summary and potential significance to field

The authors addressed all my concerns, and the manuscript is now ready for publication.

Comments for the author

The authors addressed all my concerns, and the manuscript is now ready for publication.

© 2023. Published by The Company of Biologists under the terms of the Creative Commons Attribution License (https://creativecommons.org/licenses/by/4.0/).
